# Determining antenatal medicine exposures in South African women: a comparison of three methods of ascertainment

**DOI:** 10.1186/s12884-022-04765-1

**Published:** 2022-06-03

**Authors:** Jani van der Hoven, Elizabeth Allen, Annibale Cois, Renee de Waal, Gary Maartens, Landon Myer, Thokozile Malaba, Hlengiwe Madlala, Dorothy Nyemba, Florence Phelanyane, Andrew Boulle, Ushma Mehta, Emma Kalk

**Affiliations:** 1 Division of Clinical Pharmacology, Department of Medicine, University of Cape Town, Cape Town, South Africa; 2 Division of Health Systems and Public Health, Department of Global Health, Stellenbosch University, Tygerberg, South Africa; 3 Division of Epidemiology & Biostatistics, School of Public Health & Family Medicine, University of Cape Town, Cape Town, South Africa; 4 Centre for Infectious Disease Epidemiology & Research, School of Public Health & Family Medicine, University of Cape Town, Cape Town, South Africa; 5 Provincial Health Data Centre, HealthIntelligence, Western Cape Government Health, Cape Town, South Africa

**Keywords:** Pharmacovigilance, Pregnancy, Antenatal medicine-use, Comparison of data sources, Low- and Middle-income countries

## Abstract

**Background:**

In the absence of clinical trials, data on the safety of medicine exposures in pregnancy are dependent on observational studies conducted after the agent has been licensed for use. This requires an accurate history of antenatal medicine use to determine potential risks. Medication use is commonly determined by self-report, clinician records, and electronic pharmacy data; different data sources may be more informative for different types of medication and resources may differ by setting. We compared three methods to determine antenatal medicine use (self-report, clinician records and electronic pharmacy dispensing records [EDR]) in women attending antenatal care at a primary care facility in Cape Town, South Africa in a setting with high HIV prevalence.

**Methods:**

Structured, interview-administered questionnaires recorded self-reported medicine use. Data were collected from clinician records and EDR on the same participants. We determined agreement between these data sources using Cohen’s kappa and, lacking a gold standard, used Latent Class Analysis to estimate sensitivity, specificity and positive predictive value (PPV) for each data source.

**Results:**

Between 55% and 89% of 967 women had any medicine use documented depending on the data source (median number of medicines/participant = 5 [IQR 3–6]). Agreement between the datasets was poor regardless of class except for antiretroviral therapy (ART; kappa 0.6–0.71). Overall, agreement was better between the EDR and self-report than with either dataset and the clinician records. Sensitivity and PPV were higher for self-report and the EDR and were similar for the two. Self-report was the best source for over-the-counter, traditional and complementary medicines; clinician records for vaccines and supplements; and EDR for chronic medicines.

**Conclusions:**

Medicine use in pregnancy was common and no single data source included all the medicines used. ART was the most consistently reported across all three datasets but otherwise agreement between them was poor and dependent on class. Using a single data collection method will under-estimate medicine use in pregnancy and the choice of data source should be guided by the class of the agents being investigated.

**Supplementary information:**

The online version contains supplementary material available at 10.1186/s12884-022-04765-1.

## Background

The use of prescription, over-the-counter (OTC), traditional, complementary, and alternative medicines during pregnancy is common [1]. A systematic review of out-patient prescription medicine use in high-income countries reported that between 27 and 93% of pregnant women filled at least one prescription (excluding vitamins and supplements) [1]. A similar review reported a prevalence of self-medication between 32 and 43% [2]. There are limited data on prescription medicine use during pregnancy from Africa although the literature suggests that this too is high: a prevalence of 86.9% (45.9% excluding vitamins and supplements) reported in pooled Ethiopian studies [3]; 53.5% of women in Togo [4]; and 73.2% of women in Cameroon [5]. In sub-Saharan Africa mass treatment and prevention campaigns for HIV, tuberculosis, and malaria result in widespread exposure to medicines during pregnancy.

Pregnant women should not be denied access to safe medicines at the appropriate dosages nor exposed to unsafe agents. Since pregnant women have been systematically excluded from pre-authorization pharmaceutical trials there are limited clinical trial data on the efficacy, dosing, and safety of many medicines used in pregnancy [6, 7]. Assessments of medicine safety in the mother and fetus often rely on observational studies conducted *after* the medicine has been licensed and is in regular use [6, 8]. To establish the safety profile of therapies and vaccines used in pregnancy, it is necessary to determine associations between medicine exposures and adverse pregnancy outcomes. For this, an *accurate history of medicine use during pregnancy is required*.

Antenatal medication exposure is commonly determined by self-report, clinician records, and electronic pharmacy data; different data sources may be more informative for different types of medication. Each method has strengths and limitations, and combination of all three has been recommended [9]. Such a comprehensive approach is expensive and is not feasible at scale or for on-going surveillance.

We present a comparison of three methods used to determine antenatal medicine use (self-report, clinician records, and electronic dispensing records [EDR]) in a large cohort of pregnant women presenting for antenatal care at a primary care obstetric facility in Cape Town, South Africa. We determine the contribution of each dataset to a consolidated list, the degree of agreement between datasets and whether any method offers an advantage in terms of medicine type.

## Methods

We performed a secondary analysis of data from the B-positive cohort project, a prospective study of pregnant women and their infants at a primary care maternity facility (Gugulethu Midwife Obstetric Unit [GMOU]) in Cape Town. The B-positive project aimed to comprehensively assess the effect of the World Health Organization (WHO) prevention of vertical transmission of HIV Option B + policy in the Western Cape province, South Africa. Between January 2017 and July 2018, consecutive pregnant women aged ≥ 18 years, living with and without HIV were enrolled at their first antenatal visit to GMOU. Participants attended up to three antenatal study visits depending on the gestational age at enrolment, and four post-natal study visits. At each visit, data were collected on medicine use, nutrition and food security, mental and physical health, and combination antiretroviral therapy (ART) use and adherence in women living with HIV (WLHIV). Baseline demographic and medical information was elicited at the first visit. Data were collected using standardized questionnaires by trained study field-workers and entered onto a REDCap database. Only the data on antenatal medicine use were used here. We did not assess adherence to ART or other medicines, a limitation which is noted below.

The suburb of Gugulethu has high levels of poverty and an antenatal HIV prevalence of approximately 30% [10]. GMOU is a midwife-run public sector health care facility that provides antenatal care and manages uncomplicated deliveries. If clinically indicated, women are referred to public hospitals at any stage during pregnancy or the peripartum period. Participants were enrolled at GMOU and continued follow-up regardless of referral. In South Africa, obstetric care is free at public sector facilities; most women attend at least one antenatal visit and deliver at a health care facility. Midwives are able to prescribe and dispense supplements (iron and folate), antibiotics for the treatment of urinary tract and sexually-transmitted infections and ART. In line with WHO guidelines, regular HIV screening is offered throughout pregnancy and breast-feeding. All WLHIV are initiated on life-long ART.

### Data sources

#### Self-report

Antenatal medicine use was collected by standardized interviewer-administered questionnaires at up to three visits and aimed to elicit a comprehensive report of medicine use during the preceding periods (Supplementary File 1). Women were asked to recall all prescription medication, OTC medicines and remedies, and traditional and herbal treatments. The source of medication was determined (clinic, hospital, pharmacy, grocery stores, traditional healers, spiritual healers, family and friends). Participants were asked about treatments for chronic medical conditions (e.g., HIV, hypertension, cardiac, endocrine, psychiatric conditions) and treatments for intercurrent infections (e.g., tuberculosis, sexually transmitted infections [STI], urinary tract infections.) They were asked to report on symptoms per organ system and, if present, whether they had taken any medicine or remedy to alleviate these. This combination of open-ended questions followed by specific indication-orientated and medicine-orientated enquiries has been shown to optimize response for medicine use collected at interview [11]. Medicine names and tradenames were recorded. Medicine Identification Aids with photographs of common packaging and formulations were available to the interviewers. Data from the interviews were entered into a REDCap [12, 13] database using a unique study number.

#### Clinician records

The Maternity Case Record (MCR) is a patient-held document that records all clinical consultations and investigations relating to pregnancy and delivery in the public sector in South Africa. From the first antenatal visit, the MCR documents medical conditions and current medication use elicited from the woman during the consultation by the midwife. It is updated by the attending clinician (midwife or doctor) at all subsequent visits and is retained at the site of delivery. The Western Cape Pregnancy Exposure Registry (PER) was established at GMOU in 2016 and digitized data elements from the MCR, including medicine use [14]. Registry data were entered electronically using the primary care information system which is standard in the public health facilities in the province. Women entered the Registry at their first visit to GMOU. Data were updated from the MCR after pregnancy outcome. Syndromic treatment for STI was entered from the STI register at GMOU, a paper register which documents ward-stock dispensing for vaginal discharge and genital ulcer syndromes, syphilis and vaginal candida infections. Ward stock is bulk medicine stock received by the facility; dispensing is not recorded electronically against a patient name. The Registry served as the data source for clinician records for the cohort.

#### Electronic dispensing records

The Western Cape Provincial Health Data Centre (PHDC) is a health information exchange leveraged on a unique patient identifier which is used in all public sector health services in the Western Cape province [15]. The PHDC curates dispensing data from electronic pharmacy systems (outpatient and inpatient) and was the source of the EDR. Medicines that are prescribed but not collected were not included; nor were medicines dispensed directly as ward-stock, or OTC medication. The indication for the prescription was not recorded.

The PER and PHDC are resources of the Western Cape Provincial Government and fall within its ethical and legal authority. The relevant datasets were requested and issued to the investigators under the study number; no identifiers were included.

### Anatomical therapeutic chemical classification

The Anatomical Therapeutic Chemical (ATC) classification is an international classification system maintained by the WHO which assigns an alphanumeric code to medicines [16]. There are five levels of coding describing organ system, therapeutic, pharmacological, and chemical properties. The medicines in each dataset were coded as far as possible using the ATC system. The Herbal ATC classification [17] is a similar system that codes herbal remedies by indication for use. We were unable to apply this system to the traditional and complementary products in this study as 1) the indication for use was not universally available; and 2) not all the agents contained herbal elements. For these analyses we included all traditional and complementary medicines and remedies as a single category: traditional, complementary, and alternative medication (TCAM). If there was no evidence of medicine use in a dataset, this was categorized as none.

We combined all three datasets into a Master List which provided a comprehensive record of all medicines taken per participant classified by ATC, or as TCAM or none. Each medicine appeared only once per participant regardless of how many times it was reported during pregnancy or whether it was reported in one, two, or all three datasets.

The groups within ATC level 1 are too diverse to analyze as aggregates, therefore analyses were performed at ATC level 2 (pharmacological or therapeutic subgroups) for all medicines. Agents commonly used at level 2 (i.e., > 10% in the Master List) as well as ART (J05), combination therapy for tuberculosis treatment, isoniazid (J04AC01) for tuberculosis preventive therapy (TBPT) in WLHIV, antidiabetic agents (A10) and known teratogens (e.g., anti-epileptics, psycholeptics) were analyzed at the 5^th^ ATC level. For these analyses, ATC codes less than level 5 were excluded to prevent misclassification. ART was prescribed per the South African *Guideline for the Prevention of Mother to Child Transmission of Communicable Diseases:* 1^st^ line regimen comprising a two-drug nucleotide reverse transcriptase (NRTI) backbone with a non-nucleotide reverse transcriptase inhibitor; and 2^nd^ line regimen, an NRTI backbone with a protease inhibitor [18]. ART was regarded as a single product. Based on syndromic management guidelines [19], treatment for STI was classified as metronidazole (P01AB01) alone or with/without azithromycin (J01FA10) and/or amoxicillin (J01CA04) and/or ceftriaxone (J01DD04); or ceftriaxone alone. Intramuscular benzathine penicillin (J01CE08) treatment for syphilis was classified separately. In addition, iron, folate (B03) and combination vitamin agents (A11, A12) were grouped in the single category of *vitamins and supplements*.

### Statistical analysis

Data were analyzed using STATA 15 (College Station, TX: StataCorp LP). Continuous demographic variables were summarized using medians and interquartile ranges (IQR). Categorical variables were described using proportions and compared using frequency tables. Venn diagrams graphically described the overlap between the three data sources for selected categories [20].

Cohen’s kappa with 95% CI was used to evaluate the agreement between the three datasets. Kappa values were interpreted using the Landis and Koch categories [21]: almost perfect (> 0.80), substantial (0.61 – 0.80), moderate (0.41 – 0.60), fair (0.21 – 0.40), slight (0.00 – 0.20), and poor (< 0.00). The performance of Cohen’s kappa calculations is affected by prevalence (being less reliable at low prevalence) and we also reported Prevalence and Bias-adjusted Kappa (PABAK) which assumes a prevalence of 50% and an absence of bias.

For medicine categories sufficiently represented in each of the data sources, Latent Class Analysis (LCA) was used to estimate the ‘true’ prevalence of use and the sensitivity, specificity and positive predictive value (PPV) of each data source in absence of recognized gold standard [22]. For each category, we considered a two-classes LCA model with the presence/absence of the medication in each of the three sources as observed variables. We fitted the models by penalized maximum likelihood and used the χ^2^ goodness-of-fit (GOF) test to assess the assumption of conditional independence implicit in the model. As the use of the theorical χ^2^ distribution is not warranted when data are sparse (as in our case), we applied the empirical distribution of the test statistics to calculate the *p*-value for the GOF. We obtained the empirical distribution by generating 4000 samples from the null assumption of perfect fit and computing the corresponding statistic at each iteration [23]. Estimated model parameters were used to calculate the statistics of interest and the quantified uncertainty by means and 95% CI (bootstrapped with 4000 samples). R statistical software v. 4.1 (Vienna, Austria: R Foundation for Statistical Computing) and the R package random LCA [24] were used for the LCA calculations.

### Ethical considerations

The parent and sub-studies were approved by the University of Cape Town Human Research Ethics Committee (REF 541/2015, 749/2015 and 197/2020) and the Western Cape Government Department of Health Provincial Health Research Committee (REF WC_2016RP6_286). All women provided informed consent including for access of their clinical records and linked electronic health information.

## Results

Nine-hundred and eighty-eight pregnant women were enrolled. Women who had an ectopic pregnancy (*n* = 2) or an elective termination of pregnancy before 20 weeks gestation (*n* = 2) were excluded. Seventeen women only attended a single study visit and were excluded. The final cohort comprised 967 women, 472 (48.8%) living with HIV (including six who seroconverted with HIV during the course of the pregnancy). Apart from HIV-infection, 58 (6%) women reported a chronic medical condition at enrolment, the commonest being hypertension. Seven women were treated for tuberculosis (Table 1). All medicines (excluding TCAM) were categorized to the first and second ATC levels, and 91,9% to the 5^th^ level.

**Table 1:** Maternal characteristics at enrolment (first antenatal visit)

Characteristics (*n* = 967)	
Age (years) median (IQR)	29 (25 – 34)
Age categories n (%)	
< 25 years	232 (24%)
25 – 35 years	573 (59%)
> 35 years	162 (17%)
First documented pregnancy n (%)	200 (20.7%)
Last school grade completed^a^ median (IQR)	11 (10 – 12)
Tertiary education n (%)	22 (2.3%)
Gestational age at first antenatal visit^b^ (weeks) median (IQR)	19 (13 – 24)
Living with HIV n (%)	472 (48.8%)
Chronic medical condition n (%)	58 (6%)
Diabetes mellitus	8 (0.8%)
Hypertension	27 (2.8%)
Asthma	16 (1.7%)
Epilepsy	2 (0.2%)
Cardiac disease	1 (0.1%)
Thyroid disease	6 (0.6%)
Psychiatric conditions^c^	7 (0.7%)
TB treatment during this pregnancy n (%)	7 (0.7%)
Number of medicines excl. TCAM median (IQR)	5 (3 – 6)
Number of medicine excl. TCAM & vitamins & supplements	3 (1 – 4)
Number of medicines^d^ (excl. TCAM)	
0	53 (5.5%)
1	35 (3.6%)
2	36 (3.7%)
3	182 (18.8%)
> 3	661 (68.4%)
Number of medicines^d^ (excl. TCAM & vitamins & supplements	
0	73 (7.6%)
1	191 (19.8%)
2	148 (15.3%)
3	187 (19.3%)
> 3	388 (40.1%)

*TB* Tuberculosis disease, *TCAM* Traditional, complementary and alternative medicine

^a^school grades 1 – 12; 12 is the final year of high school

^b^determined by ultrasound scan at enrolment

^c^depression, bipolar mood disorder, unknown

^d^Antiretroviral Therapy regimens were considered as a single product

**Fig.1:**
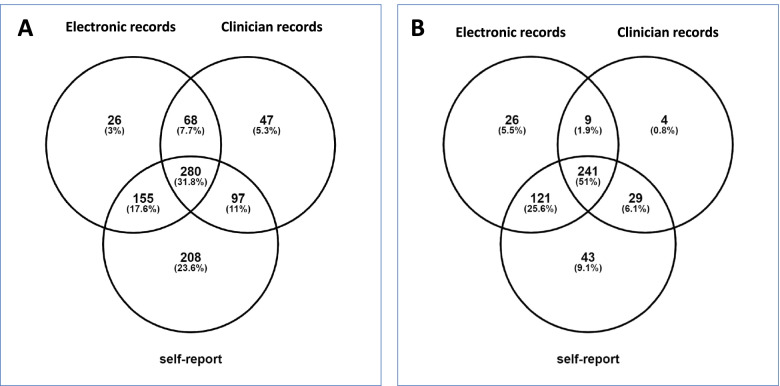
Overlap of medicine-use per data source for **A** Any medication excluding traditional, complementary and alternative medicines, and vitamins and supplements; **B** Antiretroviral therapy

Between 55 and 89% of women had any therapeutic agent use documented (i.e., prescription medication, vitamin supplements, and/or OTC medicines) depending on the data source (Fig. 1 A). When all datasets were combined, only 8 (0,8%) women had no antenatal medicines or remedies documented. When vitamins and supplements, and TCAM were excluded, 763 (78,9%) of women had evidence of medicine use in the combined Master List. Most women who used medicines during pregnancy used more than one (median 3 [IQR 1–4]) (Table 1).

TCAM were used by 220 women (22,8%) and appeared only in self-report. Vitamin and iron supplements were documented in 937 (96,9%) women, the clinician record demonstrating the highest proportion (*n* = 815; 84,3%). Other common medicines included antacids (A02; 22.1%) and analgesics (N02; 60.5%) reported mainly in self-report; and systemic antihistamines (R06; 13.13%) documented mainly in the EDR. Aspirin and non-steroidal anti-inflammatory drugs (M01) were present in both the EDR (3.83%) and self-report (4.24%). Almost one third of women (26.68%) were prescribed systemic antibiotics (J01) (Table 2). Clotrimazole cream for vaginal candidiasis (G01AF02) was present in the EDR (4.65%) and self-report (4.76%) but with little overlap (Master List 9.1%).

**Table 2:** Medicine use per dataset to Anatomical Chemical Therapeutic level 2

Total = 967		**EDR**	**EDR %**	**Clinician record**	**Clinician record %**	**Self-report**	**Self-report %**	^**a**^**Master List**	^**a**^**Master List %**
none		434	44.88%	133	13.75%	20	2.07%	8	0.82%
none ATC only (excl TCAM)		434	44.88%	133	13.75%	96	0.99%	53	5.48%
none excl. vits and TCAM		438	45.30%	475	49.10%	227	23.47%	73	7.55%
TCAM		0	0.00%	0	0.00%	220	22.75%	220	22.75%
A02	**antacids**	47	4.86%	0	0.00%	174	17.99%	204	21.10%
A03	**anticholinergics (anti-emetics)**	27	2.79%	1	0.10%	5	0.52%	33	3.41%
A06	**laxatives**	13	1.34%	12	1.24%	16	1.65%	39	4.03%
A07	**anti-diarrhoeals**	10	1.03%	2	0.21%	11	1.14%	18	1.86%
A10	**diabetic treatment**	13	1.34%	2	0.21%	4	0.41%	13	1.34%
A11	**vitamins**	179	18.51%	2	0.21%	71	7.34%	234	24.20%
A12	**mineral supplements**	3	0.31%	2	0.21%	10	1.03%	15	1.55%
B01	**anti-thrombotic agents**	1	0.10%	1	0.10%	0	0.00%	2	0.21%
B03	**anti-anaemic**	167	17.27%	815	84.28%	752	77.77%	935	96.69%
B05	**plasma expanders**	3	0.31%	0	0.00%	0	0.00%	3	0.31%
C01	**cardiac therapy**	2	0.21%	0	0.00%	0	0.00%	2	0.21%
C02	**antihypertensives**	20	2.07%	1	0.10%	4	0.41%	20	2.07%
C03	**diuretics**	16	1.65%	8	0.83%	8	0.83%	25	2.59%
C07	**B blockers**	1	0.10%	0	0.00%	0	0.00%	1	0.10%
C08	**Ca channel blockers**	8	0.83%	6	0.62%	1	0.10%	14	1.45%
C09	**ACE-inhibitors**	6	0.62%	1	0.10%	2	0.21%	8	0.83%
C10	**lipid modifying agents**	4	0.41%	1	0.10%	1	0.10%	5	0.52%
D01	**topical antifungals**	0	0.00%	0	0.00%	1	0.10%	1	0.10%
D02	**emollients & protectants**	37	3.83%	3	0.31%	25	2.59%	57	5.89%
D04	**anti-pruritics**	7	0.72%	0	0.00%	1	0.10%	8	0.83%
D06	**topical antibiotics**	4	0.41%	0	0.00%	2	0.21%	2	0.21%
D07	**topical steroids**	0	0.00%	2	0.21%	10	1.03%	26	2.69%
D08	**antiseptics**	0	0.00%	0	0.00%	1	0.10%	1	0.10%
G01	**gynae topical anti-infectives**	45	4.65%	7	0.72%	46	4.76%	88	9.10%
G02	**other gynaecologicals**	0	0.00%	0	0.00%	2	0.21%	2	0.21%
G03	**sex hormones & contraceptives**	8	0.83%	0	0.00%	14	1.45%	22	2.28%
G04	**urologicals**	6	0.62%	6	0.62%	14	1.45%	24	2.48%
H02	**systemic steroids**	9	0.93%	1	0.10%	2	0.21%	12	1.24%
H03	**thyroid therapy**	2	0.21%	0	0.00%	0	0.00%	2	0.21%
J01	**systemic antibiotics**	167	17.27%	55	5.69%	97	10.03%	258	26.68%
J02	**systemic antimycotics**	2	0.21%	0	0.00%	0	0.00%	1	0.10%
J04	**Anti-mycobacterials**	170	17.58%	5	0.52%	11	1.14%	175	18.10%
J05	**antivirals**	396	41.0%	284	29.4%	209	21.6%	450	46.5%
J07	**vaccines**	0	0.00%	225	23.27%	3	0.31%	226	23.37%
M01	**NSAID & aspirin**	37	3.83%	1	0.10%	41	4.24%	75	7.76%
N02	**analgesics**	190	19.65%	21	2.17%	505	52.22%	585	60.50%
N03	**anti-epileptics**	1	0.10%	1	0.10%	1	0.10%	2	0.21%
N05	**psycholeptics**	3	0.31%	0	0.00%	2	0.21%	4	0.41%
N06	**anti-depressants**	4	0.41%	1	0.10%	2	0.21%	7	0.72%
P01	**anti-protozoals**	36	3.70%	32	3.31%	71	7.3%	123	12.72%
P02	**anthelmintics**	3	0.31%	0	0.00%	0	0.00%	3	0.31%
P03	**ectoparasites**	13	1.34%	2	0.21%	6	0.62%	19	1.96%
R01	**nasal preparations**	5	0.52%	0	0.00%	37	3.83%	42	4.34%
R03	**Drugs for obstructive airways disease**	0	0.00%	7	0.72%	30	3.10%	38	3.93%
R05	**cough preparations**	0	0.00%	0	0.00%	40	4.14%	40	4.14%
R06	**systemic antihistamines**	54	5.58%	5	0.52%	80	8.27%	127	13.13%
S01	**ophthalmologicals**	4	0.41%	1	0.10%	1	0.10%	6	0.62%
S02	**otologicals**	1	0.10%	0	0.00%	0	0.00%	1	0.10%

*ACE* Angiotensin-II converting enzyme, *ACT* Anatomical chemical therapeutic, *NSAID* Non-steroidal anti-inflammatory drugs, *EDR* Electronic dispensing record, *TCAM* Traditional, complementary, alternative medicines

^a^The master list comprised all three data sets combined: a medicine appeared only once per participant regardless of how many times it was reported during pregnancy or whether it was reported in one, two or all three datasets

ART was documented in all WLHIV; the greatest proportion in self-report and the smallest in the clinician record (Table 3 and Fig. 1 B). Anti-mycobacterial treatments (J04; 17.3%) comprised mainly isoniazid (J04AC01) alone with seven women using combination therapy for tuberculosis. The only vaccine (23.4%) was the influenza vaccine (J07BB01), which was documented predominantly in the clinician record. Syndromic treatment for STI (13.4%) and benzathine penicillin (J01CE08) for the treatment of syphilis (3.6%) appeared most frequently in self- report (Table 3).

**Table 3:** Combination therapies and selected ATC level 5 medicine use per dataset

	**EDR**	**EDR %**	**Clinician record**	**Clinician record %**	**Self-report**	**Self-report %**	**Master List**	**Master List %**
Any vitamins and supplements^a^	173	17.9%	815	84.3%	759	78.5%	937	96.9%
Any ART^b^	397	84.1%	283	56.0%	434	91.9%	472	100%
1st line^c^	374	79.2%	265	56.1%	404	85.6%	448	94.9%
2nd line^c^	25	5.3%	18	3..8%	30	6.3%	34	7.2%
STI syndromic treatment^d^	45	4.7%	38	3.9%	73	7.6%	130	13.4%
Metronidazole (P01AB01)	36	3.7%	32	3.3%	71	7.3%	116	12.0%
Ceftriaxone (J01DD04)	0	0.0%	33	3.4%	4	0.4%	36	3.7%
Benzathine penicillin (J01CE08)^e^	0	0.0%	11	1.1%	25	2.6%	35	3.6%
Treatment for TB^f^	4	0.4%	0	0.0%	4	0.4%	7	0.7%
Isoniazid (J04AC01) as TBPT	166	17.2%	3	0.3%	7	0.7%	167	17.3%
Influenza vaccine (J07BB01)	0	0.0%	225	23.3%	3	0.3%	226	23.4%
Treatment for diabetes mellitus (A10)	13	1.3%	2	0.2%	4	0.4%	13	1.3%
Glucose lowering drugs excl. insulin^g^	13	1.3%	2	0.2%	4	0.4%	13	1.3%
Insulins (A10A)	8	0.8%	0	0.0%	0	0.0%	8	0.8%
Anti-epileptic treatments (N03)								
Phenytoin (N03AB02)	1	0.10%	1	0.10%	0	0.0%	1	0.1%
Sodium valproate (N03AG01)	0	0.0%	0	0.0%	1	0.1%	1	0.1%
Psycholeptics (N05) and anti-depressants (N06)								
Haloperidol (N05AA01)	1	0.1%	0	0.0%	0	0.0%	1	0.1%
Chlorpromazine (N05AD01)	2	0.2%	0	0.0%	0	0.0%	2	0.2%
Lithium (N05AN01)	1	0.1%	0	0.0%	0	0.0%	1	0.1%
Risperidone (N05AX08)	3	0.3%	0	0.1%	2	0.2%	4	0.4%
Fluoxetine (N06AA09)	3	0.3%	0	0.0%	1	0.1%	4	0.4%
Amitriptyline (N06AB03)	1	0.1%	1	0.1%	1	0.1%	3	0.3%
Mianserin (N06AX03)	1	0.1%	0	0.0%	0	0.0%	1	0.1%

*ACT* Anatomical chemical therapeutic, *ART* Antiretroviral therapy, *STI* Sexually transmitted infection, *TB*, Tuberculosis disease, *TBTP* TB preventive therapy, *EDR* Electronic dispensing record

^a^ Excludes vitamin B6 which is prescribed exclusively with INH as TBPT

^**b**^ in 472 WLHIV

^**c**^ Of the 472 women on ART; 1^st^ line NRTI backbone with NNRTI; 2^nd^ line NRTI backbone with PI;

10 women are represented in the total having both 1^st^ and 2^nd^ line regimens: 3 changed to 2^nd^ line treatment during pregnancy (2 in PHDC, 1 in self-report); 7 were due to discrepancies between the datasets

^**d**^metronidazole/ceftraixone ± azithromycin ± amoxicillin

^**e**^ treatment of syphilis

^**f**^ combination therapy of rifampcin + pyrazinamide + ethambutol + isoniazid

^**g**^ A10BA02 (metformin) or A10BB12 (glimepiride)

In the two women with epilepsy, phenytoin (N03AB02) and sodium valproate (N03AG01) were documented, sodium valproate only in self-report. Selective serotonin re-uptake inhibitor (SSRI) anti- depressants were recorded in five women. Risperidone (N05AX08) was used in combination with other psycholeptics in three women. (EDR; Table 3).

The agreement between each pair of datasets was determined at ATC level 2 for all medicines using Cohen’s kappa (Table 4). Generally, agreement was poor to fair (i.e., kappa < 0.40) even for agents that were commonly used (e.g., vitamins and supplements EDR vs. PER κ = 0.03; 95% CI 0.01; 0.05; EDR vs. self-report κ = 0.00; 95% CI -0.03; 0.03; PER vs self-report κ = 0.02; 95% CI-0.05; 0.08). The agreement was strongest for ART: moderate to substantial between the EDR and clinician record and substantial between self-report and the other two datasets (Table 5; Fig. 1B). For other commonly reported medicines, antacids (A02), topical gynaecological anti-infectives (exclusively clotrimazole cream for the treatment of vaginal candida infection, G01AF02), systemic antibiotics (J01), anti-mycobacterials (J04), analgesics (N02) and systemic antihistamines (R06) agreement was poor to slight (although fair for systemic antibiotics between the EDR and self- report (κ = 0.24; 95% CI 0.16; 0.32). Overall, agreement was better between the EDR and self-report than with either dataset and the clinician record.

**Table 4:** Inter-dataset agreement for ATC level 2 medications (Cohen’s kappa)

	**EDR versus Clinician Record**					**EDR versus Self-Report**					**Clinician Record versus Self-Report**				
	**kappa**	**95% CI**	**Strength**	**PABAK**	**95% CI**	**kappa**	**95% CI**	**Strength**	**PABAK**	**95% CI**	**kappa**	**95% CI**	**Strength**	**PABAK**	**95% CI**
**none**	0.33	0.27; 0.39	slight	0.33	0.27; 0.39	0.20	0.15; 0.24	slight	0.25	0.19; 0.31	0.09	0.04; 0.14	slight	0.10	0.04; 0.58
**none + TCAM**^**a**^						0.03	-0.01; 0.08	slight	0.11	0.04; 0.17	0.06	-0.01; 0.14	slight	0.58	0.53; 0.63
**A02**	none PER					0.13	0.06; 0.20	slight	0.63	0.59; 0.68					
**A03**	-0.00	0.30;-0.01	poor	0.94	0.92;0.96	-0.01	-0.02;-.00	poor	0.93	0.91;0.96	-0.00	-0.00; 0.00	poor	0.99	0.98;1.0
**A06**	-0.01	-0.02;-0.01	poor	0.95	0.93; 0.97	0.13	-0.05;0.30	slight	0.95	0.93;0.97	-0.01	-0.02;-0.01	poor	0.94	0.92;0.96
**A07**	0.16	-0.1;0.45	slight	0.98	0.97;0.99	0.37	0.11; 0.64	fair	0.97	0.96;0.99	0.15	-0.11;0.42	slight	0.98	0.96; 0.99
**A10**	0.26	-0.03; 0.56	fair	0.98	0.96;0.99	0.47	0.17; 0.76	moderate	0.98	0.97; 0.99	-0.00	-0.01; -0.00	poor	0.99	0.98; 1.00
**A11**	0.02	-0.01;0.04	slight	0.63	0.59; 0.68	0.03	-0.03;0.08	slight	0.55	0.50; 0.60	-0.00	-0.01; 0.00	poor	0.85	0.82; 0.88
**A12**	-0.00	-0.00;-0.00	poor	0.99	0.98; 1.0	-0.00	-0.01;-0.00	poor	0.97	0.96; 0.99	-0.00	-0.01;0.00	poor	0.98	0.96; 0.99
**B01**	-0.00	-0.00;-0.00	poor	1.00	0.99; 1.00	none self-report									
**B03**	0.03	0.01; 0.05	slight	-0.40	-0.46; -0.35	0.00	-0.03; 0.03	poor	-0.36	-0.42; -0.30	0.02	-0.04; 0.08	slight	0.39	0.34; 0.45
**B05**	PHDC only														
**C01**	PHDC only														
**C02**	0.09	-0.08; 0.26	slight	0.96	0.94; 0.98	0.33	0.09; 0.57	fair	0.97	0.95; 0.98	-0.00	-0.00; 0.00	poor	0.99	0.98; 1.00
**C03**	0.33	0.08; 0.57	fair	0.97	0.95; 0.98	0.16	-0.5; 0.36	slight	0.96	0.94; 0.98	0.24	-0.04; 0.53	fair	0.97	0.96; 0.99
**C07**	PHDC only														
**C08**	-0.01	-0.01; -0.00	poor	0.97	0.96; 0.99	0.22	-0.14; 0.58	fair	0.99	0.97; 1.00	-0.00	-0.00; 0.00	poor	0.99	0.97; 1.00
**C09**	0.44	0.04; 0.85	moderate	0.99	0.98; 1.00	0.25	-0.15; 0.65	fair	0.99	0.98; 1.00	0.40	-0.15; 0.94	fair	0.99	0.99; 1.00
**C10**	-0.00	-0.00; 0.00	poor	0.99	0.98; 1.00	-0.00	-0.00; 0.00	poor	0.99	0.98; 1.00	0.33	-0.15; 0.82	fair	0.99	0.98; 1.00
**D01**	self-report only														
**D02**	0.09	-0.05; 0.22	slight	0.93	0.90; 0.95	0.17	0.03; 0.30	slight	0.90	0.87; 0.92	-0.01	-0.01; 0.00	poor	0.94	0.92; 0.96
**D04**	-0.00	-0.00; 0.00	poor	0.99	0.98; 1.00	-0.00	-0.00; 0.00	poor	0.98	0.97; 0.99	-0.00	-0.00; 0.00	poor	0.99	0.98; 1.00
**D06**	self-report only														
**D07**	0.20	-0.04; 0.43	slight	0.97	0.95; 0.98	0.13	-0.05; 0.31	slight	0.95	0.93; 0.97	-0.00	-0.01; 0.00	poor	0.98	0.96; 0.99
**D08**	-0.00	-0.00; 0.00	poor			-0.00	-0.00; 0.00	poor	0.99	0.98; 1.00	-0.00	-0.00; 0.00	poor		
**G01**	0.03	-0.05; 0.10	slight	0.90	0.87; 0.92	0.11	0.01; 0.21	slight	0.84	0.81; 0.87	0.06	-0.03; 0.16	slight	0.90	0.87; 0.93
**G02**	self-report only														
**G03**	none PER					-0.01	-0.02; -0.01	poor	0.95	0.94; 0.97					
**G04**	-0.01	-0.01; -0.00	poor	0.98	0.96; 0.99	0.09	-0.10; 0.27	slight	0.96	0.95; 0.98	0.09	-0.09; 0.27	slight	0.96	0.95; 0.98
**H02**	-0.00	-0.01; 0.00	poor	0.98	0.97; 0.99	-0.00	-0.01; 0.00	poor	0.98	0.96; 0.99	-0.00	-0.00; 0.00	poor	0.99	0.9; 1.00
**H03**	PHDC only														
**J01**	0.01	-0.05; 0.06	slight	0.58	0.53; 0.63	0.24	0.16; 0.32	fair	0.64	0.59; 0.68	0.05	-0.02; 0.12	slight	0.72	0.68; 0.77
**J02**	PHDC only														
**J04**	0.02	-0.01; 0.06	slight	0.65	0.60; 0.70	0.07	0.02; 0.12	slight	0.66	0.61; 0.71	-0.01	-0.01; -0.00	poor	0.97	0.96; 0.98
**J07**	none PHDC										0.01	-0.01; 0.03	slight	0.54	0.48; 0.59
**M01**	-0.00	-0.01; 0.00	poor	0.92	0.90; 0.95	0.07	-0.03; 0.16	slight	0.86	0.82; 0.89	-0.00	-0.01; 0.00	poor	0.91	0.89; 0.94
**N02**	0.01	-0.03; 0.05	slight	0.58	0.53; 0.64	0.07	0.02; 0.12	slight	0.04	-0.02; 0.11	0.01	-0.01; 0.03	slight	-0.03	-0.09;0.03
**N03**	1.00		perfect	1.00		-0.00	-0.00; 0.00	poor	1.00	0.99; 1.00	-0.00	-0.00; 0.00	poor	1.00	0.99; 1.00
**N05**	none PER					0.40	-0.15; 0.94	fair	0.99	0.99; 1.00					
**N06**	-0.00	-0.00; 0.00	poor	0.99	0.98; 1.00	-0.00	-0.01; -0.00	poor	0.99	0.98; 1.00	-0.00	-0.00; 0.00	poor	0.99	0.99; 1.00
**P01**	0.04	-0.04; 0.13	slight	0.86	0.82; 0.89	0.12	0.03; 0.22	slight	0.80	0.77; 0.84	0.21	0.10; 0,33	fair	0.84	0.81; 0.87
**P02**	PHDC only														
**P03**	0.13	-0.10; 0.36	slight	0.97	0.96; 0.99	0.10	-0.09; .029	slight	0.96	0.95; 0.98	-0.00	-0.01; 0.00	poor	0.98	0.97; 0.99
**R01**	none PER					-0.01	-0.00; -0.02	poor	0.91	0.89; 0.94					
**R03**	0.54	0.29; 0.79	moderate	0.98	0.97; 0.99	0.21	0.04; 0.40	fair	0.93	0.90; 0.95	0.10	-0.04; 0.23	slight	0.93	0.91; 0.95
**R05**	self-report only														
**R06**	0.09	-0.01; 0.20	slight	0.89	0.86; 0.92	0.06	-0.02; 0.14	slight	0.76	0.71; 0.80	0.04	-0.02; 0.10	slight	0.83	0.80; 0.87
**S01**	-0.00	-0.00;0.00	poor	0.99	0.98; 1.00	-0.00	-0.00; 0.00	poor	0.99	0.98; 1.00	-0.00	-0.00;0.0	-0.00;0.0	1.00	0.99; 1.00
**S02**	PHDC only														

^a^Excludes TCAM and ATC medication in the self-report dataset

*ACT* Anatomical chemical therapeutic, *PABAK* Prevalence and bias-adjusted kappa, *EDR* Electronic dispensing record, *TCAM* Traditional, complementary, alternative medicines

**Table 5:** Inter-dataset agreement for combination therapies and selected ATC level 5 medications (Cohen’s kappa)

	**EDR versus Clinician Record**					**EDR versus Self-Report**					**Clinician Record versus Self-Report**				
	**kappa**	**95% CI**	**Strength**	**PABAK**	**95% CI**	**kappa**	**95% CI**	**Strength**	**PABAK**	**95% CI**	**kappa**	**95% CI**	**Strength**	**PABAK**	**95% CI**
**Any vitamins & supplements**^**a**^	0.03	0.01; 0.05	Slight	< 0.00		0.00	-0.03; 0.03	Poor	< 0.00		0.02	-0.05; 0.08	Slight	0.40	0.34; 0.45
**Any ART**	0.60	0.55; 0.65	Moderate	0.63	0.58; 0.68	0.77	0.73; 0.81	Substantial	0.78	0.74; 0.82	0.61	0.57;0.67	Substantial	0.63	0.59; 0.68
**1st line**^**b**^	0.60	0.55; 0.66	Moderate	0.65	0.60; 0.69	0.76	0.72; 0.81	Substantial	0.77	0.73; 0.81	0.61	0.56;0.67	Substantial	0.64	0.59; 0.69
**2nd line**^**b**^	0.68	0.51; 0.84	Substantial	0.98	0.96; 0.99	0.76	0.64; 0.88	Substantial	0.98	0.97; 0.99	0.62	0.45;0.78	Substantial	0.96	0.95; 0.98
**STI syndromic treatment**^**c**^	0.06	-0.04; 0.15	Slight	0.84	0.81; 0.88	0.12	0.02; 0.21	Slight	0.80	0.76; 0.84	0.23	0.12; 0.43	Fair	0.82	0.80; 0.87
**Benzathine penicillin (J01CE08)**	None					None					0.04	-0.06; 0.15	Slight	0.93	0.90; 0.95
**Treatment for TB**^**d**^	None			None		0.25	-0.15; 0.65	Fair	0.99	0.98; 1.00	None				
**INH (J04AC0) as TBPT**	0.03	0.00; 0.06	Slight	0.66	0.62; 0.71	0.06	0.01; 0.10	Slight	0.67	0.62; 0.71	0.00	-0.00; 0.00	0.98	0.97; 0.99	
**Diabetes mellitus (A10)**	0.26	-0.03; 0.56	Fair	0.98	0.96; 0.99	0.47	0.17; 0.76	Moderate	0.98	0.97; 0.99	< 0.00	-0.01; -0.00	Poor	0.99	0.97; 1.0
**Oral**^**e**^	0.26	-0.03; 0.56	Fair	0.98	0.96; 0.99	0.47	0.17; 0.76	Moderate	0.98	0.97; 0.99	< 0.00	-0.01; -0.00	Poor	0.99	0.97; 1.0
**A10A (insulins)**	< 0.00		Poor	< 0.00		< 0.00		Poor	< 0.00		< 0.00	-0.00; 0.00	Poor	< 0.00	

*ACT* Anatomical chemical therapeutic, *ART* Antiretroviral therapy, *PABAK* prevalence and bias-adjusted kappa, *INH* Isoniazid, *NNRTI* Non-nucleoside reverse transcriptase inhibitor *NRTI* Nucleoside reverse transcriptase inhibitor *EDR* Electronic dispensing record, *PI* Protease inhibitor, *STI* Sexually transmitted infection, *TB* Tuberculosis disease, *TBTP* TB preventive therapy, *TCAM* Traditional, complementary, alternative medicines

^a^ Excludes vitamin B6 which is prescribed exclusively with INH as TBPT

^b^ of the 450 women on ART; 1^st^ line NRTI backbone with NNRTI; 2.^nd^ line NRTI backbone with PI

^**c**^metronidazole/ceftraixone ± azithromycin ± amoxicillin

^**d**^ combination therapy of rifampcin + pyrazinamide + ethambutol + isoniazid

^**e**^ A10BA02 (metformin) or A10BB12 (glimepiride)

Latent class analysis was conducted for the seven medicine categories for which at least 15 records were included in each data source in order to avoid excessive sparseness with subsequent unreliability of the estimates. All models showed adequate ability to represent the observed data with no indication of significant misfit (*p*-values of the χ^2^GOF test > 0.12). For each category, Table 6 shows the estimated value of the true prevalence of medication use and sensitivity specificity and PPV of each data source in identifying the use. Sensitivity and PPV were higher for self-report and the EDR and tended to be similar for the two.

**Table 6:** True prevalence of medicine use, sensitivity, specific and positive predictive values of each data sources for selected medicine categories

				**True Prevalence [%]**		**Sensitivity [%]**		**Specificity [%]**		**Positive Predictive Value [%]**	
**Category**	**n**	**Source**	**Observed** **Prevalence [%]**	**EST**	**95% CI**	**EST**	**95% CI**	**EST**	**95% CI**	**EST**	**95% CI**
A06: Laxatives	16	Self-report	1.65	2.79	1.34; 3.83	15.63	0.02; 71.69	99.98	98.94; 99.99	95.49	97.30; 99.99
	12	Clinician record	1.24			0.01	0.01; 0.02	98.61	97.66; 99.36	0.02	0.01; 0.67
	13	EDR	1.34			12.66	0.01; 66.07	99.98	98.73; 99.99	94.50	96.28; 99.99
J01: Systemic antibiotics	97	Self-report	10.03	10.86	3.72; 24.82	48.03	23.25; 99.81	99.98	92.71; 99.99	99.67	51.22; 99.90
	55	Clinician record	5.69			9.29	2.27; 21.26	95.26	93.41; 97.26	19.27	1.09; 48.60
	167	EDR	17.27			45.41	37.04; 99.78	90.14	84.27; 99.94	35.95	0.27; 48.80
N02: Analgesics	505	Self-report	52.22	13.03	0.00; 49.43	98.70	60.18; 99.95	58.95	48.13; 99.55	26.48	0.23; 47.51
	21	Clinician record	2.17			4.98	0.26; 18.96	98.50	96.98; 99.99	33.22	0.07; 48.29
	190	EDR	19.65			35.17	20.60; 99.38	84.08	80.95; 99.82	24.87	0.25; 48.64
P01: Anti-protozoals (Metronidazole)	71	Self-report	7.34	7.34	1.34; 10.44	83.08	36.14; 99.93	98.95	94.14; 99.99	86.20	51.76; 99.87
	32	Clinician record	3.31			20.82	10.95; 67.18	98.14	97.02; 99.98	47.07	0.38; 49.32
	44	EDR	4.55			15.68	7.59; 43.35	96.37	95.02; 98.01	25.52	8.25; 47.90
ART 1.^st^ line	404	Self-report	41.78	38.57	35.57; 42.50	94.50	91.46; 97.39	94.35	91.79; 96.82	91.31	88.10; 95.58
	265	Clinician record	27.40			66.41	61.21; 71.45	99.33	98.42; 100.00	98.41	96.42; 100.00
	374	EDR	38.68			89.55	85.53; 93.24	96.19	94.07; 98.14	93.65	90.92; 97.17
ART 2.^nd^ line	30	Self-report	3.10	2.90	1.76; 4.14	86.70	72.78; 99.99	99.51	98.91; 100.00	83.97	66.65; 99.88
	18	Clinician record	1.86			59.12	39.67; 80.80	99.93	99.68; 100.00	95.93	82.53; 100.00
	26	EDR	2.69			86.69	69.45; 99.99	99.93	99.54; 100.00	97.46	83.01; 100.00
Vitamins and supplements	759	Self-report	78.49	16.13	0.00; 30.82	81.97	69.33; 96.99	23.04	11.94; 42.87	17.00	2.82; 47.77
	815	Clinician record	84.28			99.82	87.06; 99.97	22.55	15.34; 98.78	19.86	0.08; 48.23
	173	EDR	17.89			33.33	17.25; 99.11	88.89	84.20; 99.96	36.59	0.03; 48.20

*n* Number of cases reported in the source, *EST* Estimates and bounds of the 95% confidence interval. *EDR* Electronic dispensing record

## Discussion

This is one of the only reports comparing methods of ascertainment of antenatal medicine use in African women, including WLHIV. Medicine use was common, even when TCAM and vitamins and supplements were excluded (78.9%), but agreement across the three datasets assessed by Cohen’s kappa was fair to poor, even for commonly-used agents. We observed different patterns of use depending on the dataset and none provided optimal representation across all level-2 ATC categories [9]. To accommodate the heterogeneity between data sources we applied LCA to determine the sensitivity, specificity and PPV of each dataset for selected agents. In all categories tested, sensitivity was highest in self-report. The clinician record was most sensitive for vitamins and supplements but lacked sensitivity for other classes. PPV for ART was high for all three datasets, and sensitivity and specificity were similar and high for self-report and the EDR reflecting the strong agreement between the two.

The limited contribution of the clinician record could be explained by the specialist-focused structure of the South African health system: women attend specialist clinics for HIV, tuberculosis, mental health, and pre-existing hypertension and diabetes mellitus which use specific clinical stationery. Since the midwives are not involved in this care the details may not have been sought or documented in the MCR. This limitation of primary care prescribing data (i.e. the exclusion of medicines from specialist clinics, pharmacies and private doctors) has been documented previously [25]. However, in the Western Cape, HIV care in pregnancy is transferred to the antenatal facility and ART is indicated in all pregnant WLHIV [18] so the poorer representation of ART in the clinician record may reflect non-reporting and/or poor clinical record-keeping [26, 27], a limitation that should be addressed by on-going training and supervision of clinical staff.

Most antenatal medicine prescription use studies in Africa are clinical record reviews [3, 4, 28, 29] (self- report in Cameroon [5]). The data presented here suggest that dependence on this modality alone will underestimate medicine use in general and exclude certain categories completely (TCAM, anti- tuberculosis treatment, psycholeptics). Other African studies have reported use of antimalarials (prophylaxis and treatment) and anthelminthics [4, 5, 28, 30] reflecting regional burden of disease; neither of these were relevant to the urban Cape Town population described here. HIV and ART did not appear in any of the (contemporary and historical) African literature reviewed [3–5, 28, 29, 31].

Given the scale of the HIV epidemic in South Africa and the advocacy and funding that has been focused on its control, education, testing for, and treatment of HIV during pregnancy to reduce vertical transmission have been a priority. The stigma often associated with living with HIV did not prevent women from disclosing their HIV status or ART-use in self-report. ART may have a substantial impact on women’s lives and is considered significant and relevant to the health of pregnancy, which may have influenced their reporting of its use [8]. This was reflected in the high proportions of ART in each dataset and the substantial agreement between them in comparison with other agents.

European studies comparing electronic data sources with self-report found that agreement assessed by Cohen’s kappa varied according to therapeutic group, being good to very good for chronic medication for serious conditions and less reliable for occasional use agents [32, 33]. There are fewer studies comparing self-report to clinician records; in our cohort, agreement was poor to fair. In a comparison of antenatal use of medications for rheumatoid arthritis and asthma, the authors suggest that where kappa is not substantial, self-report was more reliable than medical records [34]. Norwegian studies comparing the Medical Birth Registry which is populated by clinicians during and immediately after pregnancy with the electronic Prescription Database similarly found that the sensitivity of the Registry was poor, ranging from 2–50% depending on ATC category [35]; agreement was greatest for chronic medication [27]. A validation review we conducted prior to the implementation of the PER in Cape Town in 2016 presented similar observations: the electronic database was superior to the clinician records (MCR) for chronic prescription medicines especially in women receiving care at facilities other than the MOU. Recording of ART was incomplete in the clinical stationery with errors in drug names and start and switch dates [26].

### Strengths and limitations

To our knowledge this is the only comparison of methods used to determine antenatal medicine use in Africa. We were able to assess three data sources in the same large cohort of women attending a single facility for antenatal care, including WLHIV. Given the heterogeneity of the data sources, we applied advanced statistical techniques to determine a theoretical gold standard which allowed comparison of sensitivity and specificity.

Apart from self-report, which formed part of a prospective cohort study with dedicated study materials and staff, the clinician record and EDR databases were dependent on routine programme data as recorded by the attending clinicians and we could not control for data quality. Misclassification was a potential risk. Indeed, poor clinical record-keeping may account for some of our observations. The EDR was superior to the clinician record as it consolidates information from multiple electronic sources reducing this risk [15]. However, both record dispensed medication only and may not reflect actual use. It is possible that women redeemed prescriptions but avoided use, actual consumption being recorded in the self-report in some instances. Intentional avoidance of prescription medicines in pregnancy has been described [36].

We did not report medicine use by gestational age, nor explore the use of potential teratogens by gestation as this was not the objective of the study which was a comparison of data sources. Gestational age is critical when determining potentially risky exposures as pregnancy and fetal outcomes will differ according to the timing of exposure during fetal development. Related to this, no data were presented as to whether medicines were dispensed concurrently or sequentially which would be particularly important in the case of drug-drug interactions or additive adverse maternal or teratogenic effects.

We used Cohen’s kappa as an assessment of inter-dataset agreement as the test accounts for agreement due to chance and has been used in similar analyses [32–34]. However, kappa becomes unreliable at low prevalence. We therefore used alternative methods which accounted for the heterogeneity between the datasets. Self-report proved the most sensitive for most agents tested. However, self-report is not wholly reliable being subject to recall and social desirability biases [8].

Further study is necessary to investigate patterns of medicine exposure over the course of gestation and to assess the knowledge of the risks and benefits of medicine use during pregnancy in both clinicians and women.

## Conclusions

In keeping with international reports, the different datasets offered advantages dependent on the medicines of interest. Self-report was superior for TCAM, self-medication and ART; clinician record was optimal for WHO recommended antenatal supplements and preventive medicines dispensed in primary care facilities; and the EDR for ART and treatment for other chronic medical conditions with longer recall periods, multiple and repeated dosages. Agreement between data sets was poor to fair, except for ART, and dependence on a single source may under-estimate use. In the absence of a combined data resource, the EDR presented the most accessible, reliable data.

## Supplementary Information

**Additional file 1.**

## Data Availability

The datasets generated and/or analyzed during the current study are not publicly available but may be available from the corresponding author on reasonable request.

## Abbreviations

ART: Antiretroviral Therapy
ATC: Anatomical Therapeutic Chemical classification
GOF: Goodness-of-fit test
HIV: Human Immunodeficiency Virus
IQR: Inter-quartile Range
LCA: Latent Class Analysis
MCR: Maternity Case Record
MOU: Midwife Obstetric Unit
NRTI: Nucleoside Reverse Transcriptase Inhibitor
PABAK: Prevalence and Bias-adjusted Kappa
PER: Pregnancy Exposure Registry
PHDC: Provincial Health Data Centre
PPV: Positive Predictive Value
TBPT: Tuberculosis Preventive Therapy
TCAM: Traditional, Complementary and Alternative Medicine
WHO: World Health Organization
WLHIV: Women Living with HIV
