# Supplementary material for: Determining antenatal medicine exposures in South African women: a comparison of three methods of ascertainment
Source: BMC Pregnancy Childbirth. 2022 Jun 3;22:466. doi: 10.1186/s12884-022-04765-1 (PMC9164333; doi:10.1186/s12884-022-04765-1)
Supplement: Supplementary file 1 — Additional file 1. [file 12884_2022_4765_MOESM1_ESM.pdf]

PWID: _ _ _ _ _ - _ _

MATERNAL MEDICATION USE AND SIDE EFFECT

This CRF applies to ALL enrolled BPOS Participants
To be completed at ALL Study visits

Visit Date								
D	D	M	M	M	Y	Y	Y	Y

Visit Code	
A	1

NCEDA UFUNDELE UMTHATHI NXAXHEBA

Lemibuzo ilandelayo iyakubhekisa kuwo nawaphina na amayeza namachiza othe wawasebenzisa. Oku kuquka nawaphi na amayeza kunye/okanye amachiza aphuma kubasebenzi baseklinikhi nase sibhedlela (oogqirha namanesi), ekhemesti, kwivenkile zokutya, kumagqirha, kubathandazeli, abahlobo okanye amalungu osapho. Injongo yalemibuzo kukunceda ekwenzeni uluhlu lwento yonke obuyisebenzisa **kwezinyanga zilishumi elinesibini zidlulileyo.**

Olulwazi lubalulekile kuba sifuna ukuqonda ukuba amayeza kunye/okanye namachiza obuwasebenzisa **kwezinyanga zilishumi elinesibini zidlulileyo** azakuzichaphazela na iziphumo zokukhulelwa kwakho.

Qaphela: Luninzi uphinda-phindo ukunceda wena ukuze ukhumbule onke amayeza othe wawasebenzisa!
Sicela usinyamezele kwaye uzame ukuphendula yonke imibuzo kangangoko unako.

Uyakubuzwa uluhlu lwawo onke amayeza owasebenzisayo ngoku okanye obuwasebenzisa **kwezinyanga zilishumi elinesibini zidlulileyo**. Nceda uquke onke amayeza akhutshwa ngemvume kagqirha (ART, TB, Isifo seswekile njalo-njalo), namayeza angakhutshwa ngemvume ka gqirha (i.e amayeza okanye amachiza owathenga ekhawuntarini), amayeza ongezelelweyo (e.g Vitamins), namayeza esintu kunye/okanye amayeza alinywayo kunye namachiza.

PLEASE READ OUT TO PARTICIPANT

*The following questions will refer to any medicines and/or remedies you have been taking. This includes ANY medicines and/or remedies from the clinic and hospital staff (doctors and nurses), the chemist, grocery stores, traditional healers, spiritual healers, friends or family members. The aim of this questionnaire is to help make a list of all the things you have taken **in the last 12 months.***

*This information is important because we need to understand how the medicines and/or remedies you have been taking **in the last 12 months.** to you will affect the outcome of your pregnancy.*

Please note: There is a lot of repetition to help you remember all the medications you have taken!
Please bear with us and try to answer the questions as best you can.

*You will be asked to list all medications you are currently taking or have previously used **in the last 12 months.** Please include all prescription medicines (ART, TB, and Diabetes etc.), non-prescription medicines (i.e. over the counter medicines and remedies), complementary medicines (e.g. Vitamins), traditional and/or herbal medicines and remedies.*

Initials of interviewer: ____

Table 1: IMEKO EZIKHETHEKILEYO
SPECIFIC CONDITIONS

Sizokubuza imibuzo malunga nembali yakho yezempilo.
We are going to ask you questions about your medical history.

Kwezinyanga zilishumi elinesibini zidlulileyo, ingaba ubukhe waxelelwa ngugqirha okanye ngunesi ukuba unayo nayiphi na kwenye yezimeko zezigulo zilandelayo?
In the last 12 months, *have you been told by a* ***doctor or a nurse*** *that you have ANY of the following medical conditions?*
If you did, we would like to know ***what medications and/or remedies you took for this****. These could be medications and/or remedies from a doctor, clinic, pharmacist, traditional healer, spiritual healer, supermarket/grocery store, friend or family member?*

PLEASE REMEMBER TO COMPLETE MEDICATION TABLE (TABLE 8, pg. 25) FOR EACH MEDICATION MENTIONED!!!

**Igama lemeko yesigulo**<br>***Name of medical condition***	**Yafunyaniswa nini?**<br>***When were you diagnosed?***	**Igama leyeza: Emva kokuba sithethile nawe, ingaba ubuke wasebenzisa nayo nayiphi na into malunga nalemeko?**<br>***Medicine name: Since we last spoke to you, have you taken anything for the condition?***
**1.** Ntsholongwane kagawulayo<br>*HIV*<br>☐Ewe *Yes* ☐Hayi *No*<br>→ **Ukuba HAYI Gqithela ku Q2**<br>***If NO Skip to Q2***	___ / ___ / ___<br>DD MMM YYYY	☐Ewe *Yes* ☐Hayi *No*<br>Cacisa:________<br>*Specify medication*
**2.** Isifo sephepha "TB"<br>*Tuberculosis "TB"*<br>☐Ewe *Yes* ☐Hayi *No*<br>→ **Ukuba HAYI Gqithela ku Q3**<br>***If NO Skip to Q3***	___ / ___ / ___<br>DD MMM YYYY	☐Ewe *Yes* ☐Hayi *No*<br>Cacisa:________<br>*Specify medication*
**3.** Isifo seswekile Diabetes<br>*"Sugar diabetes" Type 1 or Type 2*<br>☐Ewe *Yes* ☐Hayi *No*<br>→ **Ukuba HAYI Gqithela ku Q4**<br>***If NO Skip to Q4***	___ / ___ / ___<br>DD MMM YYYY	☐Ewe *Yes* ☐Hayi *No*<br>Cacisa:________<br>*Specify medication*
**4.** "Hi-Hi"<br>*Hypertension "High Blood Pressure"*<br>☐Ewe *Yes* ☐Hayi *No*<br>→ **Ukuba HAYI Gqithela ku Q5**<br>***If NO Skip to Q5***	___ / ___ / ___<br>DD MMM YYYY	☐Ewe *Yes* ☐Hayi *No*<br>Cacisa:________<br>*Specify medication*
**5.** Isifo sentliziyo<br>*Heart Diseases*<br>☐Ewe *Yes* ☐Hayi *No*<br>→ **Ukuba HAYI Gqithela ku Q6**<br>***If NO Skip to Q6***	___ / ___ / ___<br>DD MMM YYYY	☐Ewe *Yes* ☐Hayi *No*<br>Cacisa:________<br>*Specify medication*
**6.** Isifuba<br>*Asthma*<br>☐Ewe *Yes* ☐Hayi *No*<br>→ **Ukuba HAYI Gqithela ku Q7**<br>***If NO Skip to Q7***	___ / ___ / ___<br>DD MMM YYYY	☐Ewe *Yes* ☐Hayi *No*<br>Cacisa:________<br>*Specify medication*

PWID: _ _ _ _ _ - _ _

**7.** Isifo sokuwa<br>*Epilepsy*<br><br>☐Ewe *Yes* ☐Hayi *No*<br><br>→ **Ukuba HAYI Gqithela ku Q8**<br>***If NO Skip to Q8***	____ / ____ / ______<br>DD MMM YYYY	☐Ewe *Yes* ☐Hayi *No*<br><br>Cacisa:____________________<br>*Specify medication*
**8.** Isifo samadlala<br>*Thyroid disease*<br><br>☐Ewe *Yes* ☐Hayi *No*<br><br>→ **Ukuba HAYI Gqithela ku Q9**<br>***If NO Skip to Q9***	____ / ____ / ______<br>DD MMM YYYY	☐Ewe *Yes* ☐Hayi *No*<br><br>Cacisa:____________________<br>*Specify medication*
**9.** Enye ingulo okanye imeko yengqondo nje "ngokucinga kakhulu", uxinizelelo, ukothuka, ixhala njalo-njalo.<br>*Any psychological or mental conditions such as "thinking too much", depression, panic attacks, anxiety attacks etc."*<br><br>**NB: Efunyaniswe ngu gqirha okanye ngunesi**<br>***Diagnosed by a doctor or nurse***<br><br>☐Ewe *Yes* ☐Hayi *No*<br>→ **Ukuba HAYI Gqithela ku Q10**<br>***If NO Skip to Q10***	____ / ____ / ______<br>DD MMM YYYY	☐Ewe *Yes* ☐Hayi *No*<br><br>Cacisa:____________________<br>*Specify medication*
**10.** Ezinye naziphi na imeko zesigulo<br>*Any other medical conditions*<br><br>☐Ewe *Yes* ☐Hayi *No*<br>→ **Ukuba HAYI Gqithela ku Table2**<br>***If NO Skip to Table2***	____ / ____ / ______<br>DD MMM YYYY	☐Ewe *Yes* ☐Hayi *No*<br><br>Cacisa:____________________<br>*Specify medication*

Initials of interviewer: ____

Table 2: IMPAWU EZIDLULILEYO/ZANGOKU

PREVIOUS/CURRENT SYMPTOMS

Lemibuzo inxulumene nazo naziphi na ingxaki zempilo othe wanazo emva kokuba sithethile nawe.
*These questions relate to any health problems you may have had **since we last spoke to you.***

Kwezinyanga zilishumi elinesibini zidlulileyo, wawukhe wanayo noba yeyiphi kwezi mpawu zilandelayo? Ukuba wawukhe wanazo sifuna ukwazi ukuba leliphi iyeza okanye ichiza owalisebenzisayo? Ingangamayeza okanye amachiza akwa gqirha, eklinikhi, ekhemesti, awamagqirha, awabathandazeli, awasevenkileni yokutya, awomhlobo wakho okanye awelungu losapho lwakho?
***In the last 12 months,** did you experience ANY of the following symptoms? If you did, we would like to know what medications and/or remedies you took for this. These could be medications and/or remedies from a doctor, clinic, pharmacist, traditional healer, spiritual healer, supermarket/grocery store, friend or family member?*

PLEASE REMEMBER TO COMPLETE MEDICATION TABLE (TABLE 8, pg. 25) FOR EACH MEDICATION MENTIONED!!!

**Impawu** *Symptom*	**Ingaba ikhona into owayisebenzisayo malunga nalento?** *Did you take something for this?*	**Igama leyeza okanye lechiza** *Name of medication or remedy*	**Umhla wokuqala nowokuyeka weyeza ngalinye.** *Start and stop date for each medication used*		
**Respiratory Tract**					
Ngoku sizokubuza malunga nazo naziphi na impawu zesifuba owakhe wanazo Emva kokuba sithethile nawe. *We are now going to ask you about any respiratory or chest symptoms you could have experienced **since we last spoke to you.***					
			**Ukuqala** *Start*	**Uyokuma** *Stop*	**Iyaqhubeleka** *On-going*
**1.** Impumlo ezixineneyo *Blocked nose*<br>☐Ewe *Yes* ☐Hayi *No*<br>→ **Ukuba HAYI Gqithela ku Q2** ***If NO Skip to Q2***	☐Ewe *Yes* ☐Hayi *No*<br>→ **Ukuba HAYI Gqithela ku Q2** ***If NO Skip to Q2***	1.	__/___/___ DD MMM YYYY	__/___/___ DD MMM YYYY	☐
		2.	__/___/___ DD MMM YYYY	__/___/___ DD MMM YYYY	☐
		3.	__/___/___ DD MMM YYYY	__/___/___ DD MMM YYYY	☐
		4.	__/___/___ DD MMM YYYY	__/___/___ DD MMM YYYY	☐
**2.** Impumlo ezivuzayo *Runny nose*<br>☐Ewe *Yes* ☐Hayi *No*<br>→ **Ukuba HAYI Gqithela ku Q3** ***If NO Skip to Q3***	☐Ewe *Yes* ☐Hayi *No*<br>→ **Ukuba HAYI Gqithela ku Q3** ***If NO Skip to Q3***	1.	__/___/___ DD MMM YYYY	__/___/___ DD MMM YYYY	☐
		2.	__/___/___ DD MMM YYYY	__/___/___ DD MMM YYYY	☐
		3.	__/___/___ DD MMM YYYY	__/___/___ DD MMM YYYY	☐
		4.	__/___/___ DD MMM YYYY	__/___/___ DD MMM YYYY	☐
**3.** Ukhohlokhohlo *Cough*<br>☐Ewe *Yes* ☐Hayi *No*<br>→ **Ukuba HAYI Gqithela ku Q4** ***If NO Skip to Q4***	☐Ewe *Yes* ☐Hayi *No*<br>→ **Ukuba HAYI Gqithela ku Q4** ***If NO Skip to Q4***	1.	__/___/___ DD MMM YYYY	__/___/___ DD MMM YYYY	☐
		2.	__/___/___ DD MMM YYYY	__/___/___ DD MMM YYYY	☐
		3.	__/___/___ DD MMM YYYY	__/___/___ DD MMM YYYY	☐
		4.	__/___/___ DD MMM YYYY	__/___/___ DD MMM YYYY	☐

PWID: _ _ _ _ _ _ - _ _

Gastrointestinal Tract

Ngoku sizokubuza ngazo naziphi na impawu zesisu owawukhe wanazo **emva kokuba sithethile nawe**.
*We are now going to ask you about any stomach-related symptoms you could have experienced **since we last spoke to you**.*

**Impawu** *Symptom*	**Ingaba ikhona into owayisebenzisayo malunga nalento?** *Did you take something for this?*	**Igama leyeza okanye lechiza** *Name of medication or remedy*	**Umhla wokuqala nowokuyeka weyeza ngalinye.** *Start and stop date for each medication used*<br>**Ukuqala** *Start*	**Uyokuma** *Stop*	**Iyaqhubeleka** *On-going*
**4.** Utyatyazo *Diarrhoea*<br>☐Ewe *Yes* ☐Hayi *No*<br>→ **Ukuba HAYI Gqithela ku Q5** ***If NO Skip to Q5***	☐Ewe *Yes* ☐Hayi *No*<br>→ **Ukuba HAYI Gqithela ku Q5** ***If NO Skip to Q5***	1.	___/____/____ DD MMM YYYY	___/____/____ DD MMM YYYY	☐
		2.	___/____/____ DD MMM YYYY	___/____/____ DD MMM YYYY	☐
		3.	___/____/____ DD MMM YYYY	___/____/____ DD MMM YYYY	☐
		4.	___/____/____ DD MMM YYYY	___/____/____ DD MMM YYYY	☐
**5.** Ukugabha *Vomiting*<br>☐Ewe *Yes* ☐Hayi *No*<br>→ **Ukuba HAYI Gqithela ku Q6** ***If NO Skip to Q6***	☐Ewe *Yes* ☐Hayi *No*<br>→ **Ukuba HAYI Gqithela ku Q6** ***If NO Skip to Q6***	1.	___/____/____ DD MMM YYYY	___/____/____ DD MMM YYYY	☐
		2.	___/____/____ DD MMM YYYY	___/____/____ DD MMM YYYY	☐
		3.	___/____/____ DD MMM YYYY	___/____/____ DD MMM YYYY	☐
		4.	___/____/____ DD MMM YYYY	___/____/____ DD MMM YYYY	☐
**6.** Ukuqhinwa *Constipation*<br>☐Ewe *Yes* ☐Hayi *No*<br>→ **Ukuba HAYI Gqithela ku Q7** ***If NO Skip to Q7***	☐Ewe *Yes* ☐Hayi *No*<br>→ **Ukuba HAYI Gqithela ku Q7** ***If NO Skip to Q7***	1.	___/____/____ DD MMM YYYY	___/____/____ DD MMM YYYY	☐
		2.	___/____/____ DD MMM YYYY	___/____/____ DD MMM YYYY	☐
		3.	___/____/____ DD MMM YYYY	___/____/____ DD MMM YYYY	☐
		4.	___/____/____ DD MMM YYYY	___/____/____ DD MMM YYYY	☐
**7.** Isitshisa *Heartburn*<br>☐Ewe *Yes* ☐Hayi *No*<br>→ **Ukuba HAYI Gqithela ku Q8** ***If NO Skip to Q8***	☐Ewe *Yes* ☐Hayi *No*<br>→ **Ukuba HAYI Gqithela ku Q8** ***If NO Skip to Q8***	1.	___/____/____ DD MMM YYYY	___/____/____ DD MMM YYYY	☐
		2.	___/____/____ DD MMM YYYY	___/____/____ DD MMM YYYY	☐
		3.	___/____/____ DD MMM YYYY	___/____/____ DD MMM YYYY	☐
		4.	___/____/____ DD MMM YYYY	___/____/____ DD MMM YYYY	☐

Initials of interviewer: ____

**Impawu** *Symptom*	**Ingaba ikhona into owayisebenzisayo malunga nalento?** *Did you take something for this?*	**Igama leyeza okanye lechiza** *Name of medication or remedy*	**Umhla wokuqala nowokuyeka weyeza ngalinye.** *Start and stop date for each medication used*		
			**Ukuqala** *Start*	**Uyokuma** *Stop*	**Iyaqhubeleka** *On-going*
**8.** Isicaphucaphu *Nausea*<br>☐Ewe *Yes* ☐Hayi *No*<br>→ **Ukuba HAYI Gqithela ku Q9** ***If NO Skip to Q9***	☐Ewe *Yes* ☐Hayi *No*<br>→ **Ukuba HAYI Gqithela ku Q9** ***If NO Skip to Q9***	1.<br>2.<br>3.<br>4.	__/___/___ DD MMM YYYY<br>__/___/___ DD MMM YYYY<br>__/___/___ DD MMM YYYY<br>__/___/___ DD MMM YYYY	__/___/___ DD MMM YYYY<br>__/___/___ DD MMM YYYY<br>__/___/___ DD MMM YYYY<br>__/___/___ DD MMM YYYY	☐<br>☐<br>☐<br>☐
**9.** Ipiles *Piles or hemorrhoids*<br>☐Ewe *Yes* ☐Hayi *No*<br>→ **Ukuba HAYI Gqithela ku Q10** ***If NO Skip to Q10***	☐Ewe *Yes* ☐Hayi *No*<br>→ **Ukuba HAYI Gqithela ku Q10** ***If NO Skip to Q10***	1.<br>2.<br>3.<br>4.	__/___/___ DD MMM YYYY<br>__/___/___ DD MMM YYYY<br>__/___/___ DD MMM YYYY<br>__/___/___ DD MMM YYYY	__/___/___ DD MMM YYYY<br>__/___/___ DD MMM YYYY<br>__/___/___ DD MMM YYYY<br>__/___/___ DD MMM YYYY	☐<br>☐<br>☐<br>☐
**Genito-urinary Tract**					
Ngoku sizokubuza imibuzo malunga nempawu ezinxulumene nesinyi kunye nesibeleko sakho<br>*We are now going to ask you questions about symptoms related to your bladder and womb.*					
**10.** Umchamo otshisayo *Burning urination*<br>☐Ewe *Yes* ☐Hayi *No*<br>→ **Ukuba HAYI Gqithela ku Q11** ***If NO Skip to Q11***	☐Ewe *Yes* ☐Hayi *No*<br>→ **Ukuba HAYI Gqithela ku Q11** ***If NO Skip to Q11***	1.<br>2.<br>3.<br>4.	__/___/___ DD MMM YYYY<br>__/___/___ DD MMM YYYY<br>__/___/___ DD MMM YYYY<br>__/___/___ DD MMM YYYY	__/___/___ DD MMM YYYY<br>__/___/___ DD MMM YYYY<br>__/___/___ DD MMM YYYY<br>__/___/___ DD MMM YYYY	☐<br>☐<br>☐<br>☐
**11.** Ukuchama qho *Frequent urination*<br>☐Ewe *Yes* ☐Hayi *No*<br>→ **Ukuba HAYI Gqithela ku Q12** ***If NO Skip to Q12***	☐Ewe *Yes* ☐Hayi *No*<br>→ **Ukuba HAYI Gqithela ku Q12** ***If NO Skip to Q12***	1.<br>2.<br>3.<br>4.	__/___/___ DD MMM YYYY<br>__/___/___ DD MMM YYYY<br>__/___/___ DD MMM YYYY<br>__/___/___ DD MMM YYYY	__/___/___ DD MMM YYYY<br>__/___/___ DD MMM YYYY<br>__/___/___ DD MMM YYYY<br>__/___/___ DD MMM YYYY	☐<br>☐<br>☐<br>☐
**12.** Isinyi esibuhlungu *Bladder infection*<br>☐Ewe *Yes* ☐Hayi *No*<br>→ **Ukuba HAYI Gqithela ku Q13** ***If NO Skip to Q13***	☐Ewe *Yes* ☐Hayi *No*<br>→ **Ukuba HAYI Gqithela ku Q13** ***If NO Skip to Q13***	1.<br>2.<br>3.<br>4.	__/___/___ DD MMM YYYY<br>__/___/___ DD MMM YYYY<br>__/___/___ DD MMM YYYY<br>__/___/___ DD MMM YYYY	__/___/___ DD MMM YYYY<br>__/___/___ DD MMM YYYY<br>__/___/___ DD MMM YYYY<br>__/___/___ DD MMM YYYY	☐<br>☐<br>☐<br>☐

PWID: _ _ _ _ _ _ - _ _

**Impawu** *Symptom*	**Ingaba ikhona into owayisebenzisayo malunga nalento?** *Did you take something for this?*	**Igama leyeza okanye lechiza** *Name of medication or remedy*	**Umhla wokuqala nowokuyeka weyeza ngalinye.** *Start and stop date for each medication used*		
			**Ukuqala** *Start*	**Uyokuma** *Stop*	**Iyaqhubeleka** *On-going*
**13.** Incindi esebuntombini *Vaginal discharge*<br>☐Ewe *Yes* ☐Hayi *No*<br>→ **Ukuba HAYI Gqithela ku Q14** ***If NO Skip to Q14***	☐Ewe *Yes* ☐Hayi *No*<br>→ **Ukuba HAYI Gqithela ku Q14** ***If NO Skip to Q14***	1.<br>2.<br>3.<br>4.	__/__/__ DD MMM YYYY<br>__/__/__ DD MMM YYYY<br>__/__/__ DD MMM YYYY<br>__/__/__ DD MMM YYYY	__/__/__ DD MMM YYYY<br>__/__/__ DD MMM YYYY<br>__/__/__ DD MMM YYYY<br>__/__/__ DD MMM YYYY	☐<br>☐<br>☐<br>☐
**14.** Ukopha ebuntombini *Vaginal bleeding*<br>☐Ewe *Yes* ☐Hayi *No*<br>→ **Ukuba HAYI Gqithela ku Q15** ***If NO Skip to Q15***	☐Ewe *Yes* ☐Hayi *No*<br>→ **Ukuba HAYI Gqithela ku Q15** ***If NO Skip to Q15***	1.<br>2.<br>3.<br>4.	__/__/__ DD MMM YYYY<br>__/__/__ DD MMM YYYY<br>__/__/__ DD MMM YYYY<br>__/__/__ DD MMM YYYY	__/__/__ DD MMM YYYY<br>__/__/__ DD MMM YYYY<br>__/__/__ DD MMM YYYY<br>__/__/__ DD MMM YYYY	☐<br>☐<br>☐<br>☐
**15.** Ukurhawuzelelwa ebuntombini *Vaginal itchiness*<br>☐Ewe *Yes* ☐Hayi *No*<br>→ **Ukuba HAYI Gqithela ku Q16** ***If NO Skip to Q16***	☐Ewe *Yes* ☐Hayi *No*<br>→ **Ukuba HAYI Gqithela ku Q16** ***If NO Skip to Q16***	1.<br>2.<br>3.<br>4.	__/__/__ DD MMM YYYY<br>__/__/__ DD MMM YYYY<br>__/__/__ DD MMM YYYY<br>__/__/__ DD MMM YYYY	__/__/__ DD MMM YYYY<br>__/__/__ DD MMM YYYY<br>__/__/__ DD MMM YYYY<br>__/__/__ DD MMM YYYY	☐<br>☐<br>☐<br>☐
**General/Other**					
Ngoku sizokubuza ngengxaki othe wazifumana **emva kokuba sithethile nawe.** *We are now going to ask you about any other problems you may have experienced **since we last spoke to you**.*					
**16.** Intloko ebuhlungu *Headache*<br>☐Ewe *Yes* ☐Hayi *No*<br>→ **Ukuba HAYI Gqithela ku Q17** ***If NO Skip to Q17***	☐Ewe *Yes* ☐Hayi *No*<br>→ **Ukuba HAYI Gqithela ku Q17** ***If NO Skip to Q17***	1.<br>2.<br>3.<br>4.	__/__/__ DD MMM YYYY<br>__/__/__ DD MMM YYYY<br>__/__/__ DD MMM YYYY<br>__/__/__ DD MMM YYYY	__/__/__ DD MMM YYYY<br>__/__/__ DD MMM YYYY<br>__/__/__ DD MMM YYYY<br>__/__/__ DD MMM YYYY	☐<br>☐<br>☐<br>☐

Initials of interviewer: ____

**Impawu**<br>*Symptom*	**Ingaba ikhona into owayisebenzisayo malunga nalento?**<br>*Did you take something for this?*	**Igama leyeza okanye lechiza**<br>*Name of medication or remedy*	**Umhla wokuqala nowokuyeka weyeza ngalinye.** *Start and stop date for each medication used*<br>**Ukuqala**<br>*Start*	**Uyokuma**<br>*Stop*	**Iyaqhubeleka**<br>*On-going*
**17.** Ukurhawuzelela kolusu<br>*Itchiness of the skin*<br>☐Ewe *Yes* ☐Hayi *No*<br>→ **Ukuba HAYI Gqithela ku Q18**<br>***If NO Skip to Q18***	☐Ewe *Yes* ☐Hayi *No*<br>→ **Ukuba HAYI Gqithela ku Q18**<br>***If NO Skip to Q18***	1.<br>2.<br>3.<br>4.	___/____/____ DD MMM YYYY<br>___/____/____ DD MMM YYYY<br>___/____/____ DD MMM YYYY<br>___/____/____ DD MMM YYYY	___/____/____ DD MMM YYYY<br>___/____/____ DD MMM YYYY<br>___/____/____ DD MMM YYYY<br>___/____/____ DD MMM YYYY	☐<br>☐<br>☐<br>☐
**18.** Ukudinwa,ukutyhafa/ ukungabinamandla<br>*Fatigue/tiredness/weakness*<br>☐Ewe *Yes* ☐Hayi *No*<br>→ **Ukuba HAYI Gqithela ku Q19**<br>***If NO Skip to Q19***	☐Ewe *Yes* ☐Hayi *No*<br>→ **Ukuba HAYI Gqithela ku Q19**<br>***If NO Skip to Q19***	1.<br>2.<br>3.<br>4.	___/____/____ DD MMM YYYY<br>___/____/____ DD MMM YYYY<br>___/____/____ DD MMM YYYY<br>___/____/____ DD MMM YYYY	___/____/____ DD MMM YYYY<br>___/____/____ DD MMM YYYY<br>___/____/____ DD MMM YYYY<br>___/____/____ DD MMM YYYY	☐<br>☐<br>☐<br>☐
**19.** Ukubila ebusuku okanye ifiva<br>*Night sweats or fever*<br>☐Ewe *Yes* ☐Hayi *No*<br>→ **Ukuba HAYI Gqithela ku Q20**<br>***If NO Skip to Q20***	☐Ewe *Yes* ☐Hayi *No*<br>→ **Ukuba HAYI Gqithela ku Q20**<br>***If NO Skip to Q20***	1.<br>2.<br>3.<br>4.	___/____/____ DD MMM YYYY<br>___/____/____ DD MMM YYYY<br>___/____/____ DD MMM YYYY<br>___/____/____ DD MMM YYYY	___/____/____ DD MMM YYYY<br>___/____/____ DD MMM YYYY<br>___/____/____ DD MMM YYYY<br>___/____/____ DD MMM YYYY	☐<br>☐<br>☐<br>☐
**20.** Ukudumba kwenyawo okanye imilenze<br>*Swelling of the feet or legs*<br>☐Ewe *Yes* ☐Hayi *No*<br>→ **Ukuba HAYI Gqithela ku Q21**<br>***If NO Skip to Q21***	☐Ewe *Yes* ☐Hayi *No*<br>→ **Ukuba HAYI Gqithela ku Q21**<br>***If NO Skip to Q21***	1.<br>2.<br>3.<br>4.	___/____/____ DD MMM YYYY<br>___/____/____ DD MMM YYYY<br>___/____/____ DD MMM YYYY<br>___/____/____ DD MMM YYYY	___/____/____ DD MMM YYYY<br>___/____/____ DD MMM YYYY<br>___/____/____ DD MMM YYYY<br>___/____/____ DD MMM YYYY	☐<br>☐<br>☐<br>☐
**21.** Urhawuzelwa/ izilonda/inyebetu<br>*Rash/sores/ulcer*<br>☐Ewe *Yes* ☐Hayi *No*<br>→ **Ukuba HAYI Gqithela ku Q22**<br>***If NO Skip to Q22***	☐Ewe *Yes* ☐Hayi *No*<br>→ **Ukuba HAYI Gqithela ku Q22**<br>***If NO Skip to Q22***	1.<br>2.<br>3.<br>4.	___/____/____ DD MMM YYYY<br>___/____/____ DD MMM YYYY<br>___/____/____ DD MMM YYYY<br>___/____/____ DD MMM YYYY	___/____/____ DD MMM YYYY<br>___/____/____ DD MMM YYYY<br>___/____/____ DD MMM YYYY<br>___/____/____ DD MMM YYYY	☐<br>☐<br>☐<br>☐

Initials of interviewer:

**Impawu** *Symptom*<br><br>Ukuba **EWE,** nceda cacisa kweliphi ilungu lomzimba (e.g intlungu zamazantsi omqolo, ilungu ledolo etc.)<br>*If YES, please specify on which part of body (e.g. lower back pain, knee joint etc.)*	**Ingaba ikhona into owayisebenzisayo malunga nalento?** *Did you take something for this?*	**Igama leyeza okanye lechiza** *Name of medication or remedy*	**Umhla wokuqala nowokuyeka weyeza ngalinye.** *Start and stop date for each medication used*<br>**Ukuqala** *Start*	**Uyokuma** *Stop*	**Iyaqhubeleka** *On-going*
**22.** Enye *Other*<br><br>☐Ewe *Yes* ☐Hayi *No*<br><br>→ **Ukuba HAYI Gqithela ku Q23** ***If NO Skip to Q23***	☐Ewe *Yes* ☐Hayi *No*<br><br>→ **Ukuba HAYI Gqithela ku Q23** ***If NO Skip to Q23***	1.<br>2.<br>3.<br>4.	__/___/___ DD MMM YYYY<br>__/___/___ DD MMM YYYY<br>__/___/___ DD MMM YYYY<br>__/___/___ DD MMM YYYY	__/___/___ DD MMM YYYY<br>__/___/___ DD MMM YYYY<br>__/___/___ DD MMM YYYY<br>__/___/___ DD MMM YYYY	☐<br>☐<br>☐<br>☐

Impawu ngokubhekiselele kwintlungu
Symptoms with regards to pain

Ngoku sizokubuza ngazo naziphi na intlungu owathi wazifumana **emva kokuba sithethile nawe.**
We are now going to ask you about any pain you may have experienced ***since we last spoke to you.***

Kubalulekile ukuba ucacise kakuhle ukuba zazindawoni kanye-kanye ezontlungu.
NB: It is important that you specify where exactly the pain was.

**23.** Umqolo obuhlungu *Back Pain*<br><br>☐Ewe *Yes* ☐Hayi *No*	☐Ewe *Yes* ☐Hayi *No*<br><br>→ **Ukuba HAYI Gqithela ku Q24** ***If NO Skip to Q24***	1.<br>2.<br>3.<br>4.	__/___/___ DD MMM YYYY<br>__/___/___ DD MMM YYYY<br>__/___/___ DD MMM YYYY<br>__/___/___ DD MMM YYYY	__/___/___ DD MMM YYYY<br>__/___/___ DD MMM YYYY<br>__/___/___ DD MMM YYYY<br>__/___/___ DD MMM YYYY	☐<br>☐<br>☐<br>☐
**24.** Intlungu zamalungu *Joint pain*<br><br>☐Ewe *Yes* ☐Hayi *No*<br><br>Ukuba Ewe, Cacisa:__________<br>*If YES, Specify:*	☐Ewe *Yes* ☐Hayi *No*<br><br>→ **Ukuba HAYI Gqithela ku Q25** ***If NO Skip to Q25***	1.<br>2.<br>3.<br>4.	__/___/___ DD MMM YYYY<br>__/___/___ DD MMM YYYY<br>__/___/___ DD MMM YYYY<br>__/___/___ DD MMM YYYY	__/___/___ DD MMM YYYY<br>__/___/___ DD MMM YYYY<br>__/___/___ DD MMM YYYY<br>__/___/___ DD MMM YYYY	☐<br>☐<br>☐<br>☐
**25.** Izihlunu eziqaqambayo *Aching muscles*<br><br>☐Ewe *Yes* ☐Hayi *No*<br><br>Ukuba Ewe, Cacisa:__________<br>*If YES, Specify:*	☐Ewe *Yes* ☐Hayi *No*<br><br>→ **Ukuba HAYI Gqithela ku Q26** ***If NO Skip to Q26***	1.<br>2.<br>3.<br>4.	__/___/___ DD MMM YYYY<br>__/___/___ DD MMM YYYY<br>__/___/___ DD MMM YYYY<br>__/___/___ DD MMM YYYY	__/___/___ DD MMM YYYY<br>__/___/___ DD MMM YYYY<br>__/___/___ DD MMM YYYY<br>__/___/___ DD MMM YYYY	☐<br>☐<br>☐<br>☐

Initials of interviewer: ____

**Impawu**<br>*Symptom*	**Ingaba ikhona into owayisebenzisayo malunga nalento?**<br>*Did you take something for this?*	**Igama leyeza okanye lechiza**<br>*Name of medication or remedy*	**Umhla wokuqala nowokuyeka weyeza ngalinye.**<br>*Start and stop date for each medication used*		
			**Ukuqala**<br>*Start*	**Uyokuma**<br>*Stop*	**Iyaqhubeleka**<br>*On-going*
**26.** Ingqaqambo zomzimba<br>*Body cramps*<br><br>☐Ewe *Yes* ☐Hayi *No*<br><br>Ukuba Ewe, Cacisa:________________________<br>*If YES, Specify:*	☐Ewe *Yes* ☐Hayi *No*<br><br>→ **Ukuba HAYI Gqithela ku Q27**<br>***If NO Skip to Q27***	1.<br>2.<br>3.<br>4.	___/____/____ DD MMM YYYY<br>___/____/____ DD MMM YYYY<br>___/____/____ DD MMM YYYY<br>___/____/____ DD MMM YYYY	___/____/____ DD MMM YYYY<br>___/____/____ DD MMM YYYY<br>___/____/____ DD MMM YYYY<br>___/____/____ DD MMM YYYY	☐<br>☐<br>☐<br>☐
**27.** Ezinye intlungu<br>*Other pain*<br><br>☐Ewe *Yes* ☐Hayi *No*<br><br>Ukuba Ewe, Cacisa:________________________<br>*If YES, Specify:*	☐Ewe *Yes* ☐Hayi *No*<br><br>→ **Ukuba HAYI Gqithela ku Table 3**<br>***Skip to Table 3***	1.<br>2.<br>3.<br>4.	___/____/____ DD MMM YYYY<br>___/____/____ DD MMM YYYY<br>___/____/____ DD MMM YYYY<br>___/____/____ DD MMM YYYY	___/____/____ DD MMM YYYY<br>___/____/____ DD MMM YYYY<br>___/____/____ DD MMM YYYY<br>___/____/____ DD MMM YYYY	☐<br>☐<br>☐<br>☐

PWID: _ _ _ _ _ - _ _

Table 3: UKUSETYENZISWA KWAMAYEZA ESINTU
USE OF TRADITIONAL MEDICINE

Ngoku sizokubuza imibuzo malunga nokubonana kwakho negqirha, umthandazeli, umprofethi, umhlobo wakho okanye ilungu losapho lwakho.
We will now ask you questions about any traditional medicines which you may have received from a traditional, spiritual healer, prophet, friend or family member.

Kwezinyanga zilishumi elinesibini zidlulileyo, ubukhe wasebenzisa naliphi na iyeza okanye ichiza lesintu elokusela, elokurabha, elokurhogola, elokuhlafuna, elokufutha, elokuhlamba, elokuhambisa okanye elokugwada?
In the past 12 months, *have you used any traditional medicines and/or remedies to drink, rub, inhale, chew, steam with, wash with or "snuff" etc.?*

☐ Ewe *Yes* ☐ Hayi *No* (Ukuba HAYI, gqithela ku **Table 4**)
(*If NO, skip to* ***Table 4***)

Ukuba **EWE**, NCEDA KE UNIKEZELE NGENKCUKACHA NGEZANTSI APHA
If ***YES****, then PLEASE PROVIDE DETAILS BELOW*

PLEASE REMEMBER TO COMPLETE MEDICATION TABLE (TABLE 8, pg. 25) FOR EACH MEDICATION MENTIONED!

**Igama leyeza okanye lechiza**<br>*Medicine and/or Remedy name*	**Wawulisebenzise nini eli yeza?**<br>**Chaza Ixesha.Ukuba iyaqhubeleka yenza u (X)**<br>*When did you take this medicine? State period. If on-going (X)*	**Uhlobo olusetyenzisiweyo**<br>Nceda ukhethe kolu luhlu lulandelayo<br>Phawula **ZONKE** ezifanelekileyo<br>***Method of use***<br>*Please choose from list below. Tick* ***ALL*** *that apply*
1.	Ukuqala:<br>*Start:*<br>____ / ____ / _____<br>DD MMM YYYY<br>Uyokuma:<br>*Stop:*<br>____ / ____ / _____<br>DD MMM YYYY<br>☐ Iyaqhubeleka<br>*Ongoing*	☐ Elokuxuba nokutya *To mix with food*<br>☐ Elokusela *To drink*<br>☐ Elokuhlafuna *To chew*<br>☐ Elokuhlamba ngayo *To wash with it*<br>☐ Elokurhogola *To inhale*<br>☐ Elokuthambisa emzimbeni *To apply to the body*<br>☐ Elokufutha *To steam with*<br>☐ Elokugabha/elokuphalaza *To puke*<br>☐ Elokuhambisa *To clean the stomach with*<br>☐ Enye, *Other,* Cacisa: *Specify* ____________
2.	Ukuqala:<br>*Start:*<br>____ / ____ / _____<br>DD MMM YYYY<br>Uyokuma:<br>*Stop:*<br>____ / ____ / _____<br>DD MMM YYYY<br>☐ Iyaqhubeleka<br>*Ongoing*	☐ Elokuxuba nokutya *To mix with food*<br>☐ Elokusela *To drink*<br>☐ Elokuhlafuna *To chew*<br>☐ Elokuhlamba ngayo *To wash with it*<br>☐ Elokurhogola *To inhale*<br>☐ Elokuthambisa emzimbeni *To apply to the body*<br>☐ Elokufutha *To steam with*<br>☐ Elokugabha/elokuphalaza *To puke*<br>☐ Elokuhambisa *To clean the stomach with*<br>☐ Enye, *Other,* Cacisa: *Specify* ____________

Initials of interviewer: ____

3.	Ukuqala: *Start:* ____ / ____ / ____ DD MMM YYYY<br>Uyokuma: *Stop:* ____ / ____ / ____ DD MMM YYYY<br>☐ Iyaqhubeleka *Ongoing*	☐ Elokuxuba nokutya *To mix with food* ☐ Elokusela *To drink*<br>☐ Elokuhlafuna *To chew* ☐ Elokuhlamba ngayo *To wash with it*<br>☐ Elokurhogola *To inhale* ☐ Elokuthambisa emzimbeni *To apply to the body*<br>☐ Elokufutha *To steam with* ☐ Elokugabha/elokuphalaza *To puke*<br>☐ Elokuhambisa *To clean the stomach with*<br>☐ Enye, *Other,* Cacisa: *Specify* ____________
4.	Ukuqala: *Start:* ____ / ____ / ____ DD MMM YYYY<br>Uyokuma: *Stop:* ____ / ____ / ____ DD MMM YYYY<br>☐ Iyaqhubeleka *Ongoing*	☐ Elokuxuba nokutya *To mix with food* ☐ Elokusela *To drink*<br>☐ Elokuhlafuna *To chew* ☐ Elokuhlamba ngayo *To wash with it*<br>☐ Elokurhogola *To inhale* ☐ Elokuthambisa emzimbeni *To apply to the body*<br>☐ Elokufutha *To steam with* ☐ Elokugabha/elokuphalaza *To puke*<br>☐ Elokuhambisa *To clean the stomach with*<br>☐ Enye, *Other,* Cacisa: *Specify* ____________
5.	Ukuqala: *Start:* ____ / ____ / ____ DD MMM YYYY<br>Uyokuma: *Stop:* ____ / ____ / ____ DD MMM YYYY<br>☐ Iyaqhubeleka *Ongoing*	☐ Elokuxuba nokutya *To mix with food* ☐ Elokusela *To drink*<br>☐ Elokuhlafuna *To chew* ☐ Elokuhlamba ngayo *To wash with it*<br>☐ Elokurhogola *To inhale* ☐ Elokuthambisa emzimbeni *To apply to the body*<br>☐ Elokufutha *To steam with* ☐ Elokugabha/elokuphalaza *To puke*<br>☐ Elokuhambisa *To clean the stomach with*<br>☐ Enye, *Other,* Cacisa: *Specify* ____________
6.	Ukuqala: *Start:* ____ / ____ / ____ DD MMM YYYY<br>Uyokuma: *Stop:* ____ / ____ / ____ DD MMM YYYY<br>☐ Iyaqhubeleka *Ongoing*	☐ Elokuxuba nokutya *To mix with food* ☐ Elokusela *To drink*<br>☐ Elokuhlafuna *To chew* ☐ Elokuhlamba ngayo *To wash with it*<br>☐ Elokurhogola *To inhale* ☐ Elokuthambisa emzimbeni *To apply to the body*<br>☐ Elokufutha *To steam with* ☐ Elokugabha/elokuphalaza *To puke*<br>☐ Elokuhambisa *To clean the stomach with*<br>☐ Enye, *Other,* Cacisa: *Specify* ____________

PWID: _ _ _ _ _ - _ _

Table 4: AMAYEZA ATHENGWA EKHAWUNTARINI
OVER-THE-COUNTER MEDICATION

Kwezinyanga zilishumi elinesibini zidlulileyo, wawukhe wasebenzisa amayeza okanye amachiza ase khemesti okanye evenkileni, ukuquka ipilisi, iyeza elingamanzi, inaliti, amayeza athanjiswayo okanye nayiphi na enye into?
In the past 12 months, *did you use any medications and/or remedies from a chemist or pharmacy, or supermarket including pills, liquids, injections, ointments, creams or anything else?*

☐ Ewe *Yes* ☐ Hayi *No* (Ukuba HAYI, gqithela ku **Table 5**)
(*If NO, skip to **Table 5***)

UKUBA **EWE**, NCEDA KE UNIKEZELE NGENKCUKACHA NGEZANTSI APHA
*If **YES**, then PLEASE PROVIDE DETAILS BELOW*

PLEASE REMEMBER TO COMPLETE MEDICATION TABLE (TABLE 8, pg. 25) FOR EACH MEDICATION MENTIONED!!!

**Igama leyeza okanye elechiza**<br>*Medicine and/or Remedy name*	**Wawulisebenzisela esiphi isizathu?**<br>*What was the reason for use?*	**Waliqala nini ukuyoma nini eliyeza? Chaza Ixesha.Ukuba iyaqhubeleka yenza u (X)**<br>*When did you start and stop using this medicine? State period. If on-going (X)*
1.	Isizathu:<br>*Reason:*________________<br>________________<br>________________<br>________________	Ukuqala<br>*Start:* ___ / ___ / ____<br>DD MMM YYYY<br>Uyokuma<br>*Stop:* ___ / ___ / ____<br>DD MMM YYYY<br>☐ Iyaqhubeleka<br>*Ongoing*
2.	Isizathu:<br>*Reason:*________________<br>________________<br>________________<br>________________	Ukuqala<br>*Start:* ___ / ___ / ____<br>DD MMM YYYY<br>Uyokuma<br>*Stop:* ___ / ___ / ____<br>DD MMM YYYY<br>☐ Iyaqhubeleka<br>*Ongoing*
3.	Isizathu:<br>*Reason:*________________<br>________________<br>________________<br>________________	Ukuqala<br>*Start:* ___ / ___ / ____<br>DD MMM YYYY<br>Uyokuma<br>*Stop:* ___ / ___ / ____<br>DD MMM YYYY<br>☐ Iyaqhubeleka<br>*Ongoing*
4.	Isizathu:<br>*Reason:*________________<br>________________<br>________________<br>________________	Ukuqala<br>*Start:* ___ / ___ / ____<br>DD MMM YYYY<br>Uyokuma<br>*Stop:* ___ / ___ / ____<br>DD MMM YYYY<br>☐ Iyaqhubeleka<br>*Ongoing*

Initials of interviewer: ____

5.	Isizathu:<br>*Reason*:____________________<br>____________________<br>____________________<br>____________________	Ukuqala<br>*Start:* ___ / ___ / ____<br>DD MMM YYYY<br><br>Uyokuma<br>*Stop:* ___ / ___ / ____<br>DD MMM YYYY<br>☐ Iyaqhubeleka<br>*Ongoing*
6.	Isizathu:<br>*Reason*:____________________<br>____________________<br>____________________<br>____________________	Ukuqala<br>*Start:* ___ / ___ / ____<br>DD MMM YYYY<br><br>Uyokuma<br>*Stop:* ___ / ___ / ____<br>DD MMM YYYY<br>☐ Iyaqhubeleka<br>*Ongoing*
7.	Isizathu:<br>*Reason*:____________________<br>____________________<br>____________________<br>____________________	Ukuqala<br>*Start:* ___ / ___ / ____<br>DD MMM YYYY<br><br>Uyokuma<br>*Stop:* ___ / ___ / ____<br>DD MMM YYYY<br>☐ Iyaqhubeleka<br>*Ongoing*
8.	Isizathu:<br>*Reason*:____________________<br>____________________<br>____________________<br>____________________	Ukuqala<br>*Start:* ___ / ___ / ____<br>DD MMM YYYY<br><br>Uyokuma<br>*Stop:* ___ / ___ / ____<br>DD MMM YYYY<br>☐ Iyaqhubeleka<br>*Ongoing*
9.	Isizathu:<br>*Reason*:____________________<br>____________________<br>____________________<br>____________________	Ukuqala<br>*Start:* ___ / ___ / ____<br>DD MMM YYYY<br><br>Uyokuma<br>*Stop:* ___ / ___ / ____<br>DD MMM YYYY<br>☐ Iyaqhubeleka<br>*Ongoing*
10.	Isizathu:<br>*Reason*:____________________<br>____________________<br>____________________<br>____________________	Ukuqala<br>*Start:* ___ / ___ / ____<br>DD MMM YYYY<br><br>Uyokuma<br>*Stop:* ___ / ___ / ____<br>DD MMM YYYY<br>☐ Iyaqhubeleka<br>*Ongoing*

PWID: _ _ _ _ _ - _ _

Table 5: UKUNGENISWA/UKULALISWA ESIBHEDLELA
HOSPITAL ATTENDANCE/ADMISSIONS

**Nceda ukrobe ulwazi olunikiweyo kwi Inter-current Clinical Care CRF ulungqamanise nolu ulunikwe apha!*
**Please check that the information provided in the Inter-current Clinical Care CRF corresponds with the one provided here!*

Ngoku sizokubuza malunga nengxaki zempilo obukhe wanazo okanye onazo ngoku.
We are now going to ask you about any health problems you may have had or currently have.

Kwezinyanga zilishumi elinesibini zidlulileyo, ubukhe walalisiswa esibhedlela?
***In the past 12 months,** have you been admitted to a hospital?*

☐ Ewe *Yes* ☐ Hayi *No* (Ukuba HAYI, gqithela ku **Table 6**)
(*If NO, continue to **Table 6***)

Chaza uluhlu lwezizathu zokungeniswa/laliswa kwakho kunye namayeza othe wawafumana.
List reasons for attendance/admission and associated medications received.

PLEASE REMEMBER TO COMPLETE MEDICATION TABLE (TABLE 8, pg. 25) FOR EACH MEDICATION MENTIONED!!!

**Yintoni eyakwenza uye esibhedlela?** *What made you go to the hospital?*	**Wawungeniswe/wawulaliswe kwesiphi isibhedlela?** *In which hospital were you admitted? e.g. Mowbray, GSH*	**Wawungene/wawulele nini (ewodini) yesi sibhedlela?** *When did you attend/get admitted (in a ward) at this Hospital?*	**Igama leyeza: Ingangawo nawaphi na amayeza, ukuquka iDrips, ipilisi, isaphosithorisi kunye nenaliti.** ***Nikela ngamayeza owathi wawanikwa nokuba ZANGE ulaliswe!!!** *Medicine name: It can be any medication, including Drips, pills, suppositories and injection.* ****Provide medication given even if NOT admitted!!!***
1.	Igama lesibhedlela: *Hospital Name:*__________	____ / ____ / ____ DD MMM YYYY<br>Ixesha (iiyure/iintsuku): *Period* (hrs/days) ________	Igama lamayeza: *Medication Names:*__________
2.	Igama lesibhedlela: *Hospital Name:*__________	____ / ____ / ____ DD MMM YYYY<br>Ixesha (iiyure/iintsuku): *Period* (hrs/days) ________	Igama lamayeza: *Medication Names:*__________
3.	Igama lesibhedlela: *Hospital Name:*__________	____ / ____ / ____ DD MMM YYYY<br>Ixesha (iiyure/iintsuku): *Period* (hrs/days) ________	Igama lamayeza: *Medication Names:*__________
4.	Igama lesibhedlela: *Hospital Name:*__________	____ / ____ / ____ DD MMM YYYY<br>Ixesha (iiyure/iintsuku): *Period* (hrs/days) ________	Igama lamayeza: *Medication Names:*__________

Initials of interviewer: ____

Table 6: UKUYA EKLINIKHI
CLINIC ATTENDANCE

Ngoku sizokubuza malunga nengxaki zempilo obukhe wanazo okanye onazo ngoku.
We are now going to ask you about any health problems you may have had or currently have.

Kwezinyanga zilishumi elinesibini zidlulileyo, ubukhe watyelela eklinikhi?
***In the past 12 months,** have you visited a clinic?*

☐Ewe *Yes* ☐Hayi *No* (Ukuba HAYI, gqithela ku **Table 7**)
(*If NO, skip to **Table 7***)

Chaza uluhlu lwezizathu zokuya kwakho eklinikhi kunye namayeza othe wawafumana.
List reasons for attendance and associated medications received.

PLEASE REMEMBER TO COMPLETE MEDICATION TABLE (TABLE 8, pg. 25) FOR EACH MEDICATION MENTIONED!!!

**Yintoni eyakwenza waya eklinikhi?**<br>*What made you go to the clinic?*	**Wagqibela nini ukutyelela kule klinikhi?**<br>*When last did you visit this clinic?*	**Wawutyelele kweyiphi iklinikhi?**<br>*Which clinic did you visit?*	**Igama leyeza:**<br>**Ingangawo nawaphi na amayeza, ukuquka iDrips, ipilisi, isaphosithorisi kunye nenaliti**<br>***Medicine names:***<br>*It can be any medication, including Drips, pills, suppositories and injection.*
1.	____ / ____ / ____<br>DD MMM YYYY	Igama leklinikhi:<br>*Clinic Name:*________	Igama lamayeza:<br>*Medication Names:*________<br>________<br>________<br>________
2.	____ / ____ / ____<br>DD MMM YYYY	Igama leklinikhi:<br>*Clinic Name:*________	Igama lamayeza:<br>*Medication Names:*________<br>________<br>________<br>________
3.	____ / ____ / ____<br>DD MMM YYYY	Igama leklinikhi:<br>*Clinic Name:*________	Igama lamayeza:<br>*Medication Names:*________<br>________<br>________<br>________
4.	____ / ____ / ____<br>DD MMM YYYY	Igama leklinikhi:<br>*Clinic Name:*________	Igama lamayeza:<br>*Medication Names:*________<br>________<br>________<br>________
5.	____ / ____ / ____<br>DD MMM YYYY	Igama leklinikhi:<br>*Clinic Name:*________	Igama lamayeza:<br>*Medication Names:*________<br>________<br>________<br>________
6.	____ / ____ / ____<br>DD MMM YYYY	Igama leklinikhi:<br>*Clinic Name:*________	Igama lamayeza:<br>*Medication Names:*________<br>________<br>________<br>________

PWID: -

Table 7: UHLOBO LWAMAYEZA
SPECIFIC MEDICATIONS

Ngoku sizokubuza ukuba ubukhe wasebenzisa nayiphi na koluhlobo lwamayeza okanye amachiza
We are now going to ask you whether you have taken any of these specific medicines and/or remedies.

Kwezinyanga zilishumi elinesibini zidlulileyo, ubukhe wathatha okanye wasebenzisa nayiphi kwezi zilandelayo? Ukuba EWE, nceda *CACISA*?
In the past 12 months, *have you taken or used any of the following? If YES, please SPECIFY?*
PLEASE REMEMBER TO COMPLETE MEDICATION TABLE (TABLE 8, pg. 25) FOR EACH MEDICATION MENTIONED!!

**Uhlobo leyeza okanye Iechiza** *Specific Medicine and/ or Remedy*	**Phawula ZONKE ezifanelekileyo** *Tick ALL that Apply*	**Ulithatha kangakanani eliyeza, unyango okanye ichiza?** *How often do you take this medicine, treatment or remedy?*<br>1☐ Yonke imihla *Everyday*<br>2☐ Amaxesha 1-2 ngeveki *1-2 times per week*<br>3☐ Amaxesha 2-3 ngeveki *2-3 times per week*<br>4☐ Ngaphezu kwamaxesha amathathu ngeveki *More than 3 times per week*<br>5☐ Kanye ngenyanga *Once per month*<br>6☐ Kabini nangaphezulu ngenyanga *Twice or more times per month*<br>7☐ Kanye kuphela *Once off only*<br>8☐ Enye, Cacisa *Other, Specify*________	**Waliqala nini ukuyoma nini eliyeza? Chaza Ixesha.Ukuba iyaqhubeleka yenza u (X)** *When did you start and stop using this medicine? State period. If on-going (X)*		
		**Isihlandlo** *Frequency*	**Ukuqala** *Start*	**Uyokuma** *Stop*	**Iyaqhubelek** *On-going*
1. Ukucwangcwisa *Contraception/ Family planning*<br><br>☐Ewe *Yes*<br>☐Hayi *No*<br>→ **HAYI, Gqithela ku Q2** ***If NO, Skip to Q2***	☐Ipilisi *Pill*	1☐ 2☐ 3☐ 4☐ 5☐ 6☐ 7☐ 8☐ Cacisa, *Specify:*________	__/____/___ DD MMM YYYY	__/____/___ DD MMM YYYY	☐
	Inaliti yenyanga ezimbini- *2-month injection*	1☐ 2☐ 3☐ 4☐ 5☐ 6☐ 7☐ 8☐ Cacisa, *Specify:*________	__/____/___ DD MMM YYYY	__/____/___ DD MMM YYYY	☐
	☐Inaliti yenyanga ezintathu *3-month injection/Depo*	1☐ 2☐ 3☐ 4☐ 5☐ 6☐ 7☐ 8☐ Cacisa, *Specify:*________	__/____/___ DD MMM YYYY	__/____/___ DD MMM YYYY	☐
	☐IUD *Iluphu*	1☐ 2☐ 3☐ 4☐ 5☐ 6☐ 7☐ 8☐ Cacisa, *Specify:*________	__/____/___ DD MMM YYYY	__/____/___ DD MMM YYYY	☐
	☐Upetsho lolusu *Skin patch*	1☐ 2☐ 3☐ 4☐ 5☐ 6☐ 7☐ 8☐ Cacisa, *Specify:*________	__/____/___ DD MMM YYYY	__/____/___ DD MMM YYYY	☐
	☐Implanon	1☐ 2☐ 3☐ 4☐ 5☐ 6☐ 7☐ 8☐ Cacisa, *Specify:*________	__/____/___ DD MMM YYYY	__/____/___ DD MMM YYYY	☐
	☐Enye *Other* Casisa:________ *Specify*	1☐ 2☐ 3☐ 4☐ 5☐ 6☐ 7☐ 8☐ Cacisa, *Specify:*________ ________	__/____/___ DD MMM YYYY	__/____/___ DD MMM YYYY	☐

Initials of interviewer: ____

**Uhlobo leyeza okanye Iechiza**<br>*Specific Medicine and/ or Remedy*	**Phawula <u>ZONKE</u> ezifanelekileyo**<br>*Tick <u>ALL</u> that Apply*	**Ulithatha kangakanani eliyeza, unyango okanye ichiza?**<br>*How often do you take this medicine, treatment or remedy?*<br>1☐ Yonke imihla<br>*Everyday*<br>2☐ Amaxesha 1-2 ngeveki<br>*1-2 times per week*<br>3☐ Amaxesha 2-3 ngeveki<br>*2-3 times per week*<br>4☐ Ngaphezu kwamaxesha amathathu ngeveki<br>*More than 3 times per week*<br>5☐ Kanye ngenyanga<br>*Once per month*<br>6☐ Kabini nangaphezulu ngenyanga<br>*Twice or more times per month*<br>7☐ Kanye kuphela<br>*Once off only*<br>8☐ Enye, Cacisa<br>*Other, Specify*__________	**Waliqala nini ukuyoma nini eliyeza?**<br>**Chaza Ixesha.Ukuba iyaqhubeleka yenza u (X)**<br>*When did you start and stop using this medicine? State period. If on-going (X)*		
		**Isihlandlo**<br>*Frequency*	**Ukuqala**<br>*Start*	**Uyokuma**<br>*Stop*	**Iyaqhubelek**<br>*On-going*
2. Izakhamzimba<br>*Vitamins*<br>☐Ewe *Yes*<br>☐Hayi *No*<br>→ **HAYI, Gqithela ku Q3**<br>***If NO, Skip to Q3***	☐Vitamin B12	1☐ 2☐ 3☐ 4☐ 5☐ 6☐ 7☐ 8☐<br>Cacisa,<br>*Specify:*__________	__/____/____<br>DD MMM YYYY	__/____/____<br>DD MMM YYYY	☐
	☐Vitamin C	1☐ 2☐ 3☐ 4☐ 5☐ 6☐ 7☐ 8☐<br>Cacisa,<br>*Specify:*__________	__/____/____<br>DD MMM YYYY	__/____/____<br>DD MMM YYYY	☐
	☐Vitamin D	1☐ 2☐ 3☐ 4☐ 5☐ 6☐ 7☐ 8☐<br>Cacisa,<br>*Specify:*__________	__/____/____<br>DD MMM YYYY	__/____/____<br>DD MMM YYYY	☐
	☐Vitamin B6	1☐ 2☐ 3☐ 4☐ 5☐ 6☐ 7☐ 8☐<br>Cacisa,<br>*Specify:*__________	__/____/____<br>DD MMM YYYY	__/____/____<br>DD MMM YYYY	☐
	☐Vitamin BCo	1☐ 2☐ 3☐ 4☐ 5☐ 6☐ 7☐ 8☐<br>Cacisa,<br>*Specify:*__________	__/____/____<br>DD MMM YYYY	__/____/____<br>DD MMM YYYY	☐
	☐Enye, *other*<br>Casisa:__________<br>*Specify*	1☐ 2☐ 3☐ 4☐ 5☐ 6☐ 7☐ 8☐<br>Cacisa,<br>*Specify:*__________	__/____/____<br>DD MMM YYYY	__/____/____<br>DD MMM YYYY	☐
3. Amayeza ongezelelweyo nawokunyusa amajoni omzimba ase chemesti/evenkile ni yokutya<br>*Supplements/ Immune boosters from chemist/pharmacy/grocery store*<br>☐Ewe *Yes*<br>☐Hayi *No*<br>→ **HAYI, Gqithela ku Q4**<br>***If NO, Skip to Q4***	☐Folic acid	1☐ 2☐ 3☐ 4☐ 5☐ 6☐ 7☐ 8☐<br>Cacisa,<br>*Specify:*__________	__/____/____<br>DD MMM YYYY	__/____/____<br>DD MMM YYYY	☐
	☐Ferrous sulfate (Iron)	1☐ 2☐ 3☐ 4☐ 5☐ 6☐ 7☐ 8☐<br>Cacisa,<br>*Specify:*__________	__/____/____<br>DD MMM YYYY	__/____/____<br>DD MMM YYYY	☐
	☐Magnesium silicate	1☐ 2☐ 3☐ 4☐ 5☐ 6☐ 7☐ 8☐<br>Cacisa,<br>*Specify:*__________	__/____/____<br>DD MMM YYYY	__/____/____<br>DD MMM YYYY	☐
	☐Calcium	1☐ 2☐ 3☐ 4☐ 5☐ 6☐ 7☐ 8☐<br>Cacisa,<br>*Specify:*__________	__/____/____<br>DD MMM YYYY	__/____/____<br>DD MMM YYYY	☐
	☐Multivitamin	1☐ 2☐ 3☐ 4☐ 5☐ 6☐ 7☐ 8☐<br>Cacisa,<br>*Specify:*__________	__/____/____<br>DD MMM YYYY	__/____/____<br>DD MMM YYYY	☐
	☐Enye<br>*Other*<br>Cacisa:________<br>*Specify*	1☐ 2☐ 3☐ 4☐ 5☐ 6☐ 7☐ 8☐<br>Cacisa,<br>*Specify:*__________	__/____/____<br>DD MMM YYYY	__/____/____<br>DD MMM YYYY	☐

Initials of interviewer:

**Uhlobo leyeza okanye Iechiza** *Specific Medicine and/ or Remedy*	**Phawula ZONKE ezifanelekileyo** *Tick ALL that Apply*	**Ulithatha kangakanani eliyeza, unyango okanye ichiza?** *How often do you take this medicine, treatment or remedy?* 1☐ Yonke imihla *Everyday* 2☐ Amaxesha 1-2 ngeveki *1-2 times per week* 3☐ Amaxesha 2-3 ngeveki *2-3 times per week* 4☐ Ngaphezu kwamaxesha amathathu ngeveki *More than 3 times per week* 5☐ Kanye ngenyanga *Once per month* 6☐ Kabini nangaphezulu ngenyanga *Twice or more times per month* 7☐ Kanye kuphela *Once off only* 8☐ Enye, Cacisa *Other, Specify*________	**Waliqala nini ukuyoma nini eliyeza? Chaza Ixesha.Ukuba iyaqhubeleka yenza u (X)** *When did you start and stop using this medicine? State period. If on-going (X)*		
		**Isihlandlo** ***Frequency***	**Ukuqala** ***Start***	**Uyokuma** ***Stop***	**Iyaqhubeleka** ***On-going***
4. Iyeza lentlungu *Medicine for pain* ☐Ewe *Yes* ☐Hayi *No* → **HAYI, Gqithela ku Q5** ***If NO, Skip to Q5***	☐Paracetamol (e.g. Panado)	1☐ 2☐ 3☐ 4☐ 5☐ 6☐ 7☐ 8☐ Cacisa, *Specify:*________	__/___/__ DD MMM YYYY	__/___/__ DD MMM YYYY	☐
	☐Ibuprofen (e.g. Advil, Brufen, Nurofen)	1☐ 2☐ 3☐ 4☐ 5☐ 6☐ 7☐ 8☐ Cacisa, *Specify:*________	__/___/__ DD MMM YYYY	__/___/__ DD MMM YYYY	☐
	☐Tramadol (e.g. Tramacet)	1☐ 2☐ 3☐ 4☐ 5☐ 6☐ 7☐ 8☐ Cacisa, *Specify:*________	__/___/__ DD MMM YYYY	__/___/__ DD MMM YYYY	☐
	☐Tramal, Codeine (e.g. in Stopayne)	1☐ 2☐ 3☐ 4☐ 5☐ 6☐ 7☐ 8☐ Cacisa, *Specify:*________	__/___/__ DD MMM YYYY	__/___/__ DD MMM YYYY	☐
	☐Aspirin (e.g. grandpa)	1☐ 2☐ 3☐ 4☐ 5☐ 6☐ 7☐ 8☐ Cacisa, *Specify:*________	__/___/__ DD MMM YYYY	__/___/__ DD MMM YYYY	☐
	☐Amitriptyline (e.g. Trepilline)	1☐ 2☐ 3☐ 4☐ 5☐ 6☐ 7☐ 8☐ Cacisa, *Specify:*________	__/___/__ DD MMM YYYY	__/___/__ DD MMM YYYY	☐
	☐Enye, *Other* Cacisa:________ *Specify*	1☐ 2☐ 3☐ 4☐ 5☐ 6☐ 7☐ 8☐ Cacisa, *Specify:*________	__/___/__ DD MMM YYYY	__/___/__ DD MMM YYYY	☐

Initials of interviewer: ____

**Uhlobo leyeza okanye Iechiza**<br>*Specific Medicine and/ or Remedy*	**Phawula <u>ZONKE</u> ezifanelekileyo**<br>*Tick <u>ALL</u> that Apply*	**Ulithatha kangakanani eliyeza, unyango okanye ichiza?**<br>*How often do you take this medicine, treatment or remedy?*<br>1☐ Yonke imihla *Everyday*<br>2☐ Amaxesha 1-2 ngeveki *1-2 times per week*<br>3☐ Amaxesha 2-3 ngeveki *2-3 times per week*<br>4☐ Ngaphezu kwamaxesha amathathu ngeveki *More than 3 times per week*<br>5☐ Kanye ngenyanga *Once per month*<br>6☐ Kabini nangaphezulu ngenyanga *Twice or more times per month*<br>7☐ Kanye kuphela *Once off only*<br>8☐ Enye, Cacisa *Other, Specify*____________	**Waliqala nini ukuyoma nini eliyeza?**<br>**Chaza Ixesha.Ukuba iyaqhubeleka yenza u (X)**<br>*When did you start and stop using this medicine? State period. If on-going (X)*		
		**Isihlandlo**<br>***Frequency***	**Ukuqala**<br>***Start***	**Uyokuma**<br>***Stop***	**Iyaqhubeleka**<br>***On-going***
5. Amayeza omkhuhlane<br>*Medicines for flu*<br>☐Ewe *Yes*<br>☐Hayi *No*<br>→ **HAYI, Gqithela ku Q6**<br>***If NO Skip to Q6***	☐Cough syrup	1☐ 2☐ 3☐ 4☐ 5☐ 6☐ 7☐ 8☐<br>Cacisa,<br>*Specify:*____________	__/___/___<br>DD MMM YYYY	__/___/___<br>DD MMM YYYY	☐
	☐Antibiotics	1☐ 2☐ 3☐ 4☐ 5☐ 6☐ 7☐ 8☐<br>Cacisa,<br>*Specify:*____________	__/___/___<br>DD MMM YYYY	__/___/___<br>DD MMM YYYY	☐
	☐Chest rub	1☐ 2☐ 3☐ 4☐ 5☐ 6☐ 7☐ 8☐<br>Cacisa,<br>*Specify:*____________	__/___/___<br>DD MMM YYYY	__/___/___<br>DD MMM YYYY	☐
	☐Nasal spray	1☐ 2☐ 3☐ 4☐ 5☐ 6☐ 7☐ 8☐<br>Cacisa,<br>*Specify:*____________	__/___/___<br>DD MMM YYYY	__/___/___<br>DD MMM YYYY	☐
	☐Allergex	1☐ 2☐ 3☐ 4☐ 5☐ 6☐ 7☐ 8☐<br>Cacisa,<br>*Specify:*____________	__/___/___<br>DD MMM YYYY	__/___/___<br>DD MMM YYYY	☐
	☐ Enye, *Other*<br>Cacisa:________<br>*Specify*	1☐ 2☐ 3☐ 4☐ 5☐ 6☐ 7☐ 8☐<br>Cacisa,<br>*Specify:*____________	__/___/___<br>DD MMM YYYY	__/___/___<br>DD MMM YYYY	☐
6. Amayeza okulala<br>*Medicines for sleep*<br>☐Ewe *Yes*<br>☐Hayi *No*<br>→ **HAYI, Gqithela ku Q7**<br>***If NO Skip to Q7***	☐Amitriptyline<br>(e.g. Trepilline)	1☐ 2☐ 3☐ 4☐ 5☐ 6☐ 7☐ 8☐<br>Cacisa,<br>*Specify:*____________	__/___/___<br>DD MMM YYYY	__/___/___<br>DD MMM YYYY	☐
	☐Diazepam<br>(e.g. Valium)	1☐ 2☐ 3☐ 4☐ 5☐ 6☐ 7☐ 8☐<br>Cacisa,<br>*Specify:*____________	__/___/___<br>DD MMM YYYY	__/___/___<br>DD MMM YYYY	☐
	☐Allergex	1☐ 2☐ 3☐ 4☐ 5☐ 6☐ 7☐ 8☐<br>Cacisa,<br>*Specify:*____________	__/___/___<br>DD MMM YYYY	__/___/___<br>DD MMM YYYY	☐
	☐Enye<br>*Other*<br>Cacisa:________<br>*Specify*	1☐ 2☐ 3☐ 4☐ 5☐ 6☐ 7☐ 8☐<br>Cacisa,<br>*Specify:*____________	__/___/___<br>DD MMM YYYY	__/___/___<br>DD MMM YYYY	☐

**Uhlobo leyeza okanye Iechiza** *Specific Medicine and/ or Remedy*	**Phawula ZONKE ezifanelekileyo** *Tick ALL that Apply*	**Ulithatha kangakanani eliyeza, unyango okanye ichiza?** *How often do you take this medicine, treatment or remedy?* 1☐ Yonke imihla *Everyday* 2☐ Amaxesha 1-2 ngeveki *1-2 times per week* 3☐ Amaxesha 2-3 ngeveki *2-3 times per week* 4☐ Ngaphezu kwamaxesha amathathu ngeveki *More than 3 times per week* 5☐ Kanye ngenyanga *Once per month* 6☐ Kabini nangaphezulu ngenyanga *Twice or more times per month* 7☐ Kanye kuphela *Once off only* 8☐ Enye, Cacisa *Other, Specify*________	**Waliqala nini ukuyoma nini eliyeza? Chaza Ixesha.Ukuba iyaqhubeleka yenza u (X)** *When did you start and stop using this medicine? State period. If on-going (X)*		
		**Isihlandlo** ***Frequency***	**Ukuqala** *Start*	**Uyokuma** *Stop*	**Iyaqhubeleka** *On-going*
7. Amayeza omoya *Medicines for bloatedness* ☐Ewe *Yes* ☐Hayi *No* → **HAYI, Gqithela ku Q8** ***If NO Skip to Q8***	☐Dulcolex	1☐ 2☐ 3☐ 4☐ 5☐ 6☐ 7☐ 8☐ Cacisa, *Specify:*________	__/___/___ DD MMM YYYY	__/___/___ DD MMM YYYY	☐
	☐Buscopan	1☐ 2☐ 3☐ 4☐ 5☐ 6☐ 7☐ 8☐ Cacisa, *Specify:*________	__/___/___ DD MMM YYYY	__/___/___ DD MMM YYYY	☐
	☐Eno	1☐ 2☐ 3☐ 4☐ 5☐ 6☐ 7☐ 8☐ Cacisa, *Specify:*________	__/___/___ DD MMM YYYY	__/___/___ DD MMM YYYY	☐
	☐Enye *Other* Cacisa:_____ *Specify*	1☐ 2☐ 3☐ 4☐ 5☐ 6☐ 7☐ 8☐ Cacisa, *Specify:*________	__/___/___ DD MMM YYYY	__/___/___ DD MMM YYYY	☐
8. Isitshisa *Heartburn* ☐Ewe *Yes* ☐Hayi *No* → **HAYI, Gqithela ku Q9** ***If NO Skip to Q9***	☐Eno	1☐ 2☐ 3☐ 4☐ 5☐ 6☐ 7☐ 8☐ Cacisa, *Specify:*________	__/___/___ DD MMM YYYY	__/___/___ DD MMM YYYY	☐
	☐Gaviscon	1☐ 2☐ 3☐ 4☐ 5☐ 6☐ 7☐ 8☐ Cacisa, *Specify:*________	__/___/___ DD MMM YYYY	__/___/___ DD MMM YYYY	☐
	☐Rennies	1☐ 2☐ 3☐ 4☐ 5☐ 6☐ 7☐ 8☐ Cacisa, *Specify:*________	__/___/___ DD MMM YYYY	__/___/___ DD MMM YYYY	☐
	☐Ranitidine(e.g. Zantac)	1☐ 2☐ 3☐ 4☐ 5☐ 6☐ 7☐ 8☐ Cacisa, *Specify:*________	__/___/___ DD MMM YYYY	__/___/___ DD MMM YYYY	☐
	☐Brown sugar	1☐ 2☐ 3☐ 4☐ 5☐ 6☐ 7☐ 8☐ Cacisa, *Specify:*________	__/___/___ DD MMM YYYY	__/___/___ DD MMM YYYY	☐
	☐Enye *Other* Cacisa:_____ *Specify*	1☐ 2☐ 3☐ 4☐ 5☐ 6☐ 7☐ 8☐ Cacisa, *Specify:*________	__/___/___ DD MMM YYYY	__/___/___ DD MMM YYYY	☐

Initials of interviewer: ____

**Uhlobo leyeza okanye Iechiza**<br>*Specific Medicine and/ or Remedy*	**Phawula ZONKE ezifanelekileyo**<br>*Tick ALL that Apply*	**Ulithatha kangakanani eliyeza, unyango okanye ichiza?**<br>*How often do you take this medicine, treatment or remedy?*<br>1☐ Yonke imihla *Everyday*<br>2☐ Amaxesha 1-2 ngeveki *1-2 times per week*<br>3☐ Amaxesha 2-3 ngeveki *2-3 times per week*<br>4☐ Ngaphezu kwamaxesha amathathu ngeveki *More than 3 times per week*<br>5☐ Kanye ngenyanga *Once per month*<br>6☐ Kabini nangaphezulu ngenyanga *Twice or more times per month*<br>7☐ Kanye kuphela *Once off only*<br>8☐ Enye, Cacisa *Other, Specify*__________	**Waliqala nini ukuyoma nini eliyeza?**<br>**Chaza Ixesha.Ukuba iyaqhubeleka yenza u (X)**<br>*When did you start and stop using this medicine? State period. If on-going (X)*		
		**Isihlandlo**<br>***Frequency***	**Ukuqala**<br>*Start*	**Uyokuma**<br>*Stop*	**Iyaqhubeleka**<br>*On-going*
9. Incindi emdaka ephuma ebuntombini<br>*Vaginal discharge/itchiness*<br><br>☐Ewe *Yes*<br>☐Hayi *No*<br>→ **HAYI, Gqithela ku Q10**<br>***If NO Skip to Q10***	☐Metronidazole (e.g. Flagyl)	1☐ 2☐ 3☐ 4☐ 5☐ 6☐ 7☐ 8☐<br>Cacisa,<br>*Specify:*__________	__/___/___<br>DD MMM YYYY	__/___/___<br>DD MMM YYYY	☐
	☐Penicillin injection	1☐ 2☐ 3☐ 4☐ 5☐ 6☐ 7☐ 8☐<br>Cacisa,<br>*Specify:*__________	__/___/___<br>DD MMM YYYY	__/___/___<br>DD MMM YYYY	☐
	☐Ceftriaxone (stat- dose)	1☐ 2☐ 3☐ 4☐ 5☐ 6☐ 7☐ 8☐<br>Cacisa,<br>*Specify:*__________	__/___/___<br>DD MMM YYYY	__/___/___<br>DD MMM YYYY	☐
	☐Clotrimazole cream (e.g. Medaspor, Candizole)	1☐ 2☐ 3☐ 4☐ 5☐ 6☐ 7☐ 8☐<br>Cacisa,<br>*Specify:*__________	__/___/___<br>DD MMM YYYY	__/___/___<br>DD MMM YYYY	☐
	☐Enye *Other*<br>Cacisa:________<br>*Specify*	1☐ 2☐ 3☐ 4☐ 5☐ 6☐ 7☐ 8☐<br>Cacisa,<br>*Specify:*__________	__/___/___<br>DD MMM YYYY	__/___/___<br>DD MMM YYYY	☐

PWID: -

**Uhlobo leyeza okanye Iechiza** *Specific Medicine and/ or Remedy*	**Phawula ZONKE ezifanelekileyo** *Tick ALL that Apply*	**Ulithatha kangakanani eliyeza, unyango okanye ichiza?** *How often do you take this medicine, treatment or remedy?* 1☐ Yonke imihla *Everyday* 2☐ Amaxesha 1-2 ngeveki *1-2 times per week* 3☐ Amaxesha 2-3 ngeveki *2-3 times per week* 4☐ Ngaphezu kwamaxesha amathathu ngeveki *More than 3 times per week* 5☐ Kanye ngenyanga *Once per month* 6☐ Kabini nangaphezulu ngenyanga *Twice or more times per month* 7☐ Kanye kuphela *Once off only* 8☐ Enye, Cacisa *Other, Specify*____	**Waliqala nini ukuyoma nini eliyeza?** **Chaza Ixesha.Ukuba iyaqhubeleka yenza u (X)** *When did you start and stop using this medicine? State period. If on-going (X)*		
		**Isihlandlo** *Frequency*	**Ukuqala** *Start*	**Uyokuma** *Stop*	**Iyaqhubeleka** *On-going*
10. Amayeza okanye amachiza asezirhoxweni e.g etuckshophu/spaza/ kubathengisi basezitratweni/ ezitendini *Medicines/remedies from an informal trader? e.g. tuck-shop/spaza/street hawkers* ☐Ewe *Yes* ☐Hayi *No* → **HAYI, Gqithela ku Q11** ***If NO Skip to Q11***	☐Impepho	1☐ 2☐ 3☐ 4☐ 5☐ 6☐ 7☐ 8☐ Cacisa, *Specify:*____	__/___/__ DD MMM YYYY	__/___/__ DD MMM YYYY	☐
	☐Amafutha enj'olwandle	1☐ 2☐ 3☐ 4☐ 5☐ 6☐ 7☐ 8☐ Cacisa, *Specify:*____	__/___/__ DD MMM YYYY	__/___/__ DD MMM YYYY	☐
	☐Rasta's "medium crods"	1☐ 2☐ 3☐ 4☐ 5☐ 6☐ 7☐ 8☐ Cacisa, *Specify:*____	__/___/__ DD MMM YYYY	__/___/__ DD MMM YYYY	☐
	☐Clay stones	1☐ 2☐ 3☐ 4☐ 5☐ 6☐ 7☐ 8☐ Cacisa, *Specify:*____	__/___/__ DD MMM YYYY	__/___/__ DD MMM YYYY	☐
	☐ Umchamo wemfene	1☐ 2☐ 3☐ 4☐ 5☐ 6☐ 7☐ 8☐ Cacisa, *Specify:*____	__/___/__ DD MMM YYYY	__/___/__ DD MMM YYYY	☐
	☐Enye *Other* Cacisa:____ *Specify*	1☐ 2☐ 3☐ 4☐ 5☐ 6☐ 7☐ 8☐ Cacisa, *Specify:*____	__/___/__ DD MMM YYYY	__/___/__ DD MMM YYYY	☐

Initials of interviewer: ____

<table>
<tr><td>Uhlobo leyeza okanye Iechiza
Specific Medicine and/ or Remedy</td><td>Phawula <u>ZONKE</u> ezifanelekileyo
Tick <u>ALL</u> that Apply</td><td>Ulithatha kangakanani eliyeza, unyango okanye ichiza?
How often do you take this medicine, treatment or remedy?
1☐ Yonke imihla Everyday
2☐ Amaxesha 1-2 ngeveki 1-2 times per week
3☐ Amaxesha 2-3 ngeveki 2-3 times per week
4☐ Ngaphezu kwamaxesha amathathu ngeveki More than 3 times per week
5☐ Kanye ngenyanga Once per month
6☐ Kabini nangaphezulu ngenyanga Twice or more times per month
7☐ Kanye kuphela Once off only
8☐ Enye, Cacisa Other, Specify__________</td><td colspan="3">Waliqala nini ukuyoma nini eliyeza?
Chaza Ixesha.Ukuba iyaqhubeleka yenza u (X)
When did you start and stop using this medicine? State period. If on-going (X)</td></tr>
<tr><td colspan="2"></td><td>Isihlandlo
Frequency</td><td>Ukuqala
Start</td><td>Uyokuma
Stop</td><td>Iyaqhubeleka
On-going</td></tr>
<tr><td rowspan="4">11. Ingaba ikhona into oyisebenzisayo kwezi zidweliswe kwikholam elandelayo ngasekhohlo kwakho? Are you taking anything listed in the next column to your right?

☐Ewe Yes
☐Hayi No

→ HAYI, Gqithela ku Table 8
If NO Skip to Table 8</td><td>☐Bactrim</td><td>1☐ 2☐ 3☐ 4☐ 5☐ 6☐ 7☐ 8☐
Cacisa,
Specify:__________</td><td>__/____/___
DD MMM YYYY</td><td>__/____/___
DD MMM YYYY</td><td>☐</td></tr>
<tr><td>☐INH</td><td>1☐ 2☐ 3☐ 4☐ 5☐ 6☐ 7☐ 8☐
Cacisa,
Specify:__________</td><td>__/____/___
DD MMM YYYY</td><td>__/____/___
DD MMM YYYY</td><td>☐</td></tr>
<tr><td>☐Druppels (e.g. Lennon's or Herman's)</td><td>1☐ 2☐ 3☐ 4☐ 5☐ 6☐ 7☐ 8☐
Cacisa,
Specify:__________</td><td>__/____/___
DD MMM YYYY</td><td>__/____/___
DD MMM YYYY</td><td>☐</td></tr>
<tr><td>☐Enye
Other
Cacisa:________
Specify</td><td>1☐ 2☐ 3☐ 4☐ 5☐ 6☐ 7☐ 8☐
Cacisa,
Specify:__________</td><td>__/____/___
DD MMM YYYY</td><td>__/____/___
DD MMM YYYY</td><td>☐</td></tr>
</table>

PWID: _ _ _ _ _ _ - _ _

Table 8: INGCOMBOLO ZOLUHLU LWAMAYEZA
DETAILED MEDICATION LIST

Olu luluhlu **lwamayeza namachiza obuwasebenzisa emva kokuba sithethile nawe**. Ndizakuphinda ndijonge olu luhlu lwamayeza kwakhona ndikunye nawe ukuqinisekisa ukuba yonke into ilungile.
*This is a list of the **medicines and remedies you have taken since we last spoke to you.** I would like to go through this list again with you just to make sure that everything is correct.*

Nceda undazise ukuba kukho impazamo okanye ukuba ikhona into oyikhumbulayo esingayikhankanyanga
Please let me know if there is a mistake or if you remember something we have not mentioned.

PLEASE REMEMBER TO COMPLETE THIS MEDICATION TABLE FOR EACH MEDICATION MENTIONED EARLIER!

**Igama leyeza** ***Name of medication***	**Wawulifumene phi iyeza elo? Khetha ikhowudi efanelekileyo** ***From where was the medication received?***	**Waqala nini kwaye wayeka nini ukusebenzisa iyeza elo? Chaza ixesha. Ukuba uyaqhubeleka (phawula)** ***When did you start and stop using the medication?*** *State period. If on-going (X).*	**Sithini isizathu sokuyeka kwakho ukulisebenzisa iyeza elo? Khetha efanelekileyo** ***What was the reason/s for stopping use?***	**Wawukhe wayifumana nayiphi na imiphumela yalo? Ukuba EWE, nceda ucacise ukuba wasebenzisa ntoni ukuyinceda lonto.** ***Did you experience any side effects?*** *If YES, please specify what you took for it*
	☐ Ugqirha *Doctor*<br>☐ Unesi *Nurse*<br>☐ Khemesti *Chemist*<br>☐ Igqirha *Traditional Healer*<br>☐ Ivenkile yokutya *Grocery store*<br>☐ Enye *Other*<br><br>Ukuba kukho enye into nceda ucacise:<br>*If other please specify*:<br>______________	Ukuqala:<br>*Start:*<br>____ / ____ / _____<br>DD MMM YYYY<br><br>Uyokuma:<br>*Stop:*<br>____ / ____ / _____<br>DD MMM YYYY<br><br>☐ Iyaqhubeleka<br>*Ongoing*	☐ Ndabangcono<br>*Felt better*<br>☐ Landiphelela<br>*Ran out*<br>☐ Ndaligqiba<br>*Completed course*<br>☐ Ndaxelelwa ukuba ndiliyeke<br>*Told to stop*<br>Waxelelwa ngubani ukuba liyeke?<br>*If "told to stop" please say by whom*<br>______________<br><br>☐ Lalingasebenzi<br>*Was not working*<br>☐ Imiphumela<br>*Side effects*<br>☐ Enye<br>*Other*<br>Cacisa: ______________<br>*Specify*	☐ Ewe *Yes*<br>Cacisa:__________<br>*Specify:*<br><br>☐ Hayi *No*

**Igama leyeza**<br>***Name of medication***	**Wawulifumene phi iyeza elo? Khetha ikhowudi efanelekileyo**<br>***From where was the medication received?***	**Waqala nini kwaye wayeka nini ukusebenzisa iyeza elo?**<br>**Chaza ixesha.**<br>**Ukuba uyaqhubeleka (phawula)**<br>***When did you start and stop using the medication?***<br>*State period. If on-going (X)*	**Sithini isizathu sokuyeka kwakho ukulisebenzisa iyeza elo?**<br>**Khetha efanelekileyo**<br>***What was the reason/s for stopping use?***	**Wawukhe wayifumana nayiphi na imiphumela yalo?**<br>**Ukuba EWE, nceda ucacise ukuba wasebenzisa ntoni ukuyinceda lonto.**<br>***Did you experience any side effects?***<br>*If YES, please specify what you took for it*
	☐ Ugqirha *Doctor*<br>☐ Unesi *Nurse*<br>☐ Khemesti *Chemist*<br>☐ Igqirha *Traditional Healer*<br>☐ Ivenkile yokutya<br>*Grocery store*<br>☐ Enye *Other*<br><br>Ukuba kukho enye into nceda ucacise:<br>*If other please specify*:<br>____________________	Ukuqala:<br>*Start:*<br>____ / ____ / _____<br>DD MMM YYYY<br><br>Uyokuma:<br>*Stop:*<br>____ / ____ / _____<br>DD MMM YYYY<br><br>☐ Iyaqhubeleka<br>*Ongoing*	☐ Ndabangcono<br>*Felt better*<br>☐ Landiphelela<br>*Ran out*<br>☐ Ndaligqiba<br>*Completed course*<br>☐ Ndaxelelwa ukuba ndiliyeke<br>*Told to stop*<br>Waxelelwa ngubani ukuba liyeke?<br>*If "told to stop" please say by whom*<br>________________<br>☐ Lalingasebenzi<br>*Was not working*<br>☐ Imiphumela<br>*Side effects*<br>☐ Enye<br>*Other*<br>Cacisa: ______________<br>*Specify*	☐ Ewe *Yes*<br>Cacisa:__________<br>*Specify:*<br><br>☐ Hayi *No*
	☐ Ugqirha *Doctor*<br>☐ Unesi *Nurse*<br>☐ Khemesti *Chemist*<br>☐ Igqirha *Traditional Healer*<br>☐ Ivenkile yokutya<br>*Grocery store*<br>☐ Enye *Other*<br><br>Ukuba kukho enye into nceda ucacise:<br>*If other please specify*:<br>____________________	Ukuqala:<br>*Start:*<br>____ / ____ / _____<br>DD MMM YYYY<br><br>Uyokuma:<br>*Stop:*<br>____ / ____ / _____<br>DD MMM YYYY<br><br>☐ Iyaqhubeleka<br>*Ongoing*	☐ Ndabangcono<br>*Felt better*<br>☐ Landiphelela<br>*Ran out*<br>☐ Ndaligqiba<br>*Completed course*<br>☐ Ndaxelelwa ukuba ndiliyeke<br>*Told to stop*<br>Waxelelwa ngubani ukuba liyeke?<br>*If "told to stop" please say by whom*<br>________________<br>☐ Lalingasebenzi<br>*Was not working*<br>☐ Imiphumela<br>*Side effects*<br>☐ Enye<br>*Other*<br>Cacisa: ______________<br>*Specify*	☐ Ewe *Yes*<br>Cacisa:__________<br>*Specify:*<br><br>☐ Hayi *No*

Initials of interviewer:

PWID: __ __ __ __ __ - __ __

**Igama leyeza**<br>***Name of medication***	**Wawulifumene phi iyeza elo? Khetha ikhowudi efanelekileyo**<br>***From where was the medication received?***	**Waqala nini kwaye wayeka nini ukusebenzisa iyeza elo? Chaza ixesha. Ukuba uyaqhubeleka (phawula)**<br>***When did you start and stop using the medication?***<br>*State period. If on-going (X)*	**Sithini isizathu sokuyeka kwakho ukulisebenzisa iyeza elo? Khetha efanelekileyo**<br>***What was the reason/s for stopping use?***	**Wawukhe wayifumana nayiphi na imiphumela yalo? Ukuba EWE, nceda ucacise ukuba wasebenzisa ntoni ukuyinceda lonto.**<br>***Did you experience any side effects?***<br>*If YES, please specify what you took for it*
	☐ Ugqirha *Doctor*<br>☐ Unesi *Nurse*<br>☐ Khemesti *Chemist*<br>☐ Igqirha *Traditional Healer*<br>☐ Ivenkile yokutya *Grocery store*<br>☐ Enye *Other*<br>Ukuba kukho enye into nceda ucacise:<br>*If other please specify*:<br>________________	Ukuqala:<br>*Start:*<br>____ / ____ / _____<br>DD MMM YYYY<br>Uyokuma:<br>*Stop:*<br>____ / ____ / _____<br>DD MMM YYYY<br>☐ Iyaqhubeleka<br>*Ongoing*	☐ Ndabangcono *Felt better*<br>☐ Landiphelela *Ran out*<br>☐ Ndaligqiba *Completed course*<br>☐ Ndaxelelwa ukuba ndiliyeke *Told to stop*<br>Waxelelwa ngubani ukuba liyeke?<br>*If "told to stop" please say by whom*<br>______________<br>☐ Lalingasebenzi *Was not working*<br>☐ Imiphumela *Side effects*<br>☐ Enye *Other*<br>Cacisa: __________<br>*Specify*	☐ Ewe *Yes*<br>Cacisa:________<br>*Specify:*<br>☐ Hayi *No*
	☐ Ugqirha *Doctor*<br>☐ Unesi *Nurse*<br>☐ Khemesti *Chemist*<br>☐ Igqirha *Traditional Healer*<br>☐ Ivenkile yokutya *Grocery store*<br>☐ Enye *Other*<br>Ukuba kukho enye into nceda ucacise:<br>*If other please specify*:<br>________________	Ukuqala:<br>*Start:*<br>____ / ____ / _____<br>DD MMM YYYY<br>Uyokuma:<br>*Stop:*<br>____ / ____ / _____<br>DD MMM YYYY<br>☐ Iyaqhubeleka<br>*Ongoing*	☐ Ndabangcono *Felt better*<br>☐ Landiphelela *Ran out*<br>☐ Ndaligqiba *Completed course*<br>☐ Ndaxelelwa ukuba ndiliyeke *Told to stop*<br>Waxelelwa ngubani ukuba liyeke?<br>*If "told to stop" please say by whom*<br>______________<br>☐ Lalingasebenzi *Was not working*<br>☐ Imiphumela *Side effects*<br>☐ Enye *Other*<br>Cacisa: __________<br>*Specify*	☐ Ewe *Yes*<br>Cacisa:________<br>*Specify:*<br>☐ Hayi *No*

Initials of interviewer: ____

**Igama leyeza**<br>***Name of medication***	**Wawulifumene phi iyeza elo? Khetha ikhowudi efanelekileyo**<br>***From where was the medication received?***	**Waqala nini kwaye wayeka nini ukusebenzisa iyeza elo? Chaza ixesha. Ukuba uyaqhubeleka (phawula)**<br>***When did you start and stop using the medication?***<br>*State period. If on-going (X)*	**Sithini isizathu sokuyeka kwakho ukulisebenzisa iyeza elo? Khetha efanelekileyo**<br>***What was the reason/s for stopping use?***	**Wawukhe wayifumana nayiphi na imiphumela yalo? Ukuba EWE, nceda ucacise ukuba wasebenzisa ntoni ukuyinceda lonto.**<br>***Did you experience any side effects?***<br>*If YES, please specify what you took for it*
	☐ Ugqirha *Doctor*<br>☐ Unesi *Nurse*<br>☐ Khemesti *Chemist*<br>☐ Igqirha *Traditional Healer*<br>☐ Ivenkile yokutya *Grocery store*<br>☐ Enye *Other*<br><br>Ukuba kukho enye into nceda ucacise:<br>*If other please specify*:<br>________________________	Ukuqala:<br>*Start:*<br>____ / ____ / ____<br>DD MMM YYYY<br><br>Uyokuma:<br>*Stop:*<br>____ / ____ / ____<br>DD MMM YYYY<br><br>☐ Iyaqhubeleka<br>*Ongoing*	☐ Ndabangcono *Felt better*<br>☐ Landiphelela *Ran out*<br>☐ Ndaligqiba *Completed course*<br>☐ Ndaxelelwa ukuba ndiliyeke *Told to stop*<br>Waxelelwa ngubani ukuba liyeke?<br>*If "told to stop" please say by whom*<br>____________________<br>☐ Lalingasebenzi *Was not working*<br>☐ Imiphumela *Side effects*<br>☐ Enye *Other*<br>Cacisa: _______________<br>*Specify*	☐ Ewe *Yes*<br>Cacisa:____________<br>*Specify:*<br><br>☐ Hayi *No*
	☐ Ugqirha *Doctor*<br>☐ Unesi *Nurse*<br>☐ Khemesti *Chemist*<br>☐ Igqirha *Traditional Healer*<br>☐ Ivenkile yokutya *Grocery store*<br>☐ Enye *Other*<br><br>Ukuba kukho enye into nceda ucacise:<br>*If other please specify*:<br>________________________	Ukuqala:<br>*Start:*<br>____ / ____ / ____<br>DD MMM YYYY<br><br>Uyokuma:<br>*Stop:*<br>____ / ____ / ____<br>DD MMM YYYY<br><br>☐ Iyaqhubeleka<br>*Ongoing*	☐ Ndabangcono *Felt better*<br>☐ Landiphelela *Ran out*<br>☐ Ndaligqiba *Completed course*<br>☐ Ndaxelelwa ukuba ndiliyeke *Told to stop*<br>Waxelelwa ngubani ukuba liyeke?<br>*If "told to stop" please say by whom*<br>____________________<br>☐ Lalingasebenzi *Was not working*<br>☐ Imiphumela *Side effects*<br>☐ Enye *Other*<br>Cacisa: _______________<br>*Specify*	☐ Ewe *Yes*<br>Cacisa:____________<br>*Specify:*<br><br>☐ Hayi *No*

PWID: __ __ __ __ __ - __ __

**Igama leyeza** *Name of medication*	**Wawulifumene phi iyeza elo? Khetha ikhowudi efanelekileyo** *From where was the medication received?*	**Waqala nini kwaye wayeka nini ukusebenzisa iyeza elo? Chaza ixesha. Ukuba uyaqhubeleka (phawula)** *When did you start and stop using the medication? State period. If on-going (X)*	**Sithini isizathu sokuyeka kwakho ukulisebenzisa iyeza elo? Khetha efanelekileyo** *What was the reason/s for stopping use?*	**Wawukhe wayifumana nayiphi na imiphumela yalo? Ukuba EWE, nceda ucacise ukuba wasebenzisa ntoni ukuyinceda lonto.** *Did you experience any side effects? If YES, please specify what you took for it*
	☐ Ugqirha *Doctor*<br>☐ Unesi *Nurse*<br>☐ Khemesti *Chemist*<br>☐ Igqirha *Traditional Healer*<br>☐ Ivenkile yokutya *Grocery store*<br>☐ Enye *Other*<br>Ukuba kukho enye into nceda ucacise: *If other please specify*:<br>________________	Ukuqala: *Start:*<br>____ / ____ / ____<br>DD MMM YYYY<br>Uyokuma: *Stop:*<br>____ / ____ / ____<br>DD MMM YYYY<br>☐ Iyaqhubeleka *Ongoing*	☐ Ndabangcono *Felt better*<br>☐ Landiphelela *Ran out*<br>☐ Ndaligqiba *Completed course*<br>☐ Ndaxelelwa ukuba ndiliyeke *Told to stop*<br>Waxelelwa ngubani ukuba liyeke? *If "told to stop" please say by whom*<br>______________<br>☐ Lalingasebenzi *Was not working*<br>☐ Imiphumela *Side effects*<br>☐ Enye *Other*<br>Cacisa: __________ *Specify*	☐ Ewe *Yes*<br>Cacisa:________ *Specify:*<br>☐ Hayi *No*
	☐ Ugqirha *Doctor*<br>☐ Unesi *Nurse*<br>☐ Khemesti *Chemist*<br>☐ Igqirha *Traditional Healer*<br>☐ Ivenkile yokutya *Grocery store*<br>☐ Enye *Other*<br>Ukuba kukho enye into nceda ucacise: *If other please specify*:<br>________________	Ukuqala: *Start:*<br>____ / ____ / ____<br>DD MMM YYYY<br>Uyokuma: *Stop:*<br>____ / ____ / ____<br>DD MMM YYYY<br>☐ Iyaqhubeleka *Ongoing*	☐ Ndabangcono *Felt better*<br>☐ Landiphelela *Ran out*<br>☐ Ndaligqiba *Completed course*<br>☐ Ndaxelelwa ukuba ndiliyeke *Told to stop*<br>Waxelelwa ngubani ukuba liyeke? *If "told to stop" please say by whom*<br>______________<br>☐ Lalingasebenzi *Was not working*<br>☐ Imiphumela *Side effects*<br>☐ Enye *Other*<br>Cacisa: __________ *Specify*	☐ Ewe*Yes*<br>Cacisa:________ *Specify:*<br>☐ Hayi *No*

Initials of interviewer: ____

**Igama leyeza** *Name of medication*	**Wawulifumene phi iyeza elo? Khetha ikhowudi efanelekileyo** *From where was the medication received?*	**Waqala nini kwaye wayeka nini ukusebenzisa iyeza elo? Chaza ixesha. Ukuba uyaqhubeleka (phawula)** *When did you start and stop using the medication?* *State period. If on-going (X)*	**Sithini isizathu sokuyeka kwakho ukulisebenzisa iyeza elo? Khetha efanelekileyo** *What was the reason/s for stopping use?*	**Wawukhe wayifumana nayiphi na imiphumela yalo? Ukuba EWE, nceda ucacise ukuba wasebenzisa ntoni ukuyinceda lonto.** *Did you experience any side effects?* *If YES, please specify what you took for it*
	☐ Ugqirha *Doctor*<br>☐ Unesi *Nurse*<br>☐ Khemesti *Chemist*<br>☐ Igqirha *Traditional Healer*<br>☐ Ivenkile yokutya *Grocery store*<br>☐ Enye *Other*<br><br>Ukuba kukho enye into nceda ucacise:<br>*If other please specify*:<br>______________________	Ukuqala:<br>*Start:*<br>____ / ____ / ____<br>DD MMM YYYY<br><br>Uyokuma:<br>*Stop:*<br>____ / ____ / ____<br>DD MMM YYYY<br><br>☐ Iyaqhubeleka *Ongoing*	☐ Ndabangcono *Felt better*<br>☐ Landiphelela *Ran out*<br>☐ Ndaligqiba *Completed course*<br>☐ Ndaxelelwa ukuba ndiliyeke *Told to stop*<br><br>Waxelelwa ngubani ukuba liyeke? *If "told to stop" please say by whom*<br>__________________<br>☐ Lalingasebenzi *Was not working*<br>☐ Imiphumela *Side effects*<br>☐ Enye *Other*<br>Cacisa: ______________<br>*Specify*	☐ Ewe *Yes*<br><br>Cacisa:____________________<br>*Specify:*<br><br>☐ Hayi *No*
	☐ Ugqirha *Doctor*<br>☐ Unesi *Nurse*<br>☐ Khemesti *Chemist*<br>☐ Igqirha *Traditional Healer*<br>☐ Ivenkile yokutya *Grocery store*<br>☐ Enye *Other*<br><br>Ukuba kukho enye into nceda ucacise:<br>*If other please specify*:<br>______________________	Ukuqala:<br>*Start:*<br>____ / ____ / ____<br>DD MMM YYYY<br><br>Uyokuma:<br>*Stop:*<br>____ / ____ / ____<br>DD MMM YYYY<br><br>☐ Iyaqhubeleka *Ongoing*	☐ Ndabangcono *Felt better*<br>☐ Landiphelela *Ran out*<br>☐ Ndaligqiba *Completed course*<br>☐ Ndaxelelwa ukuba ndiliyeke *Told to stop*<br><br>Waxelelwa ngubani ukuba liyeke? *If "told to stop" please say by whom*<br>__________________<br>☐ Lalingasebenzi *Was not working*<br>☐ Imiphumela *Side effects*<br>☐ Enye *Other*<br>Cacisa: ______________<br>*Specify*	☐ Ewe *Yes*<br><br>Cacisa:____________________<br>*Specify:*<br><br>☐ Hayi *No*

PWID: _ _ _ _ _ - _ _

***<u>Isikhumbuzo</u>**

NCEDA UZE NEKHADI LAKHO LASE KLINIKI OKANYE NAMAYEZA KUNYE NAMACHIZA AKHO KWIXESHA ELIZAYO SIKUBONA. SICELA USIPHATHELE IQOKOBHE UKUBA AMAYEZA AKHO APHELILE.

****<u>Reminder:</u>***

PLEASE COULD YOU BRING ALONG YOUR CLINIC CARD OR YOUR MEDICINES AND REMEDIES WITH YOU NEXT TIME WE SEE YOU. KINDLY ALSO BRING ALONG EMPTY MEDICINE CONTAINERS IF YOUR MEDICINES ARE FINISHED.

Initials of interviewer: ____

NOTES:

Please write notes and/or any other comments here:

Signed Interviewer completing CRF: ______________________ Date: ____ / ______ / _____
DD MMM YYYY

Signed QC Officer: ______________ Date: ____ / ______ / _____
DD MMM YYYY

Signed Study Coordinator: ______________ Date: ____ / ______ / _____
DD MMM YYYY
